# The spike-timing-dependent plasticity of VIP interneurons in motor cortex

**DOI:** 10.3389/fncel.2024.1389094

**Published:** 2024-04-19

**Authors:** Amanda R. McFarlan, Connie Guo, Isabella Gomez, Chaim Weinerman, Tasha A. Liang, P. Jesper Sjöström

**Affiliations:** ^1^Centre for Research in Neuroscience, BRaIN Program, Department of Neurology and Neurosurgery, Research Institute of the McGill University Health Centre, Montreal General Hospital, Montreal, QC, Canada; ^2^Integrated Program in Neuroscience, McGill University, Montreal, QC, Canada

**Keywords:** VIP, inhibitory interneurons, plasticity, spike-timing-dependent plasticity, motor cortex

## Abstract

The plasticity of inhibitory interneurons (INs) plays an important role in the organization and maintenance of cortical microcircuits. Given the many different IN types, there is an even greater diversity in synapse-type-specific plasticity learning rules at excitatory to excitatory (E→I), I→E, and I→I synapses. I→I synapses play a key disinhibitory role in cortical circuits. Because they typically target other INs, vasoactive intestinal peptide (VIP) INs are often featured in I→I→E disinhibition, which upregulates activity in nearby excitatory neurons. VIP IN dysregulation may thus lead to neuropathologies such as epilepsy. In spite of the important activity regulatory role of VIP INs, their long-term plasticity has not been described. Therefore, we characterized the phenomenology of spike-timing-dependent plasticity (STDP) at inputs and outputs of genetically defined VIP INs. Using a combination of whole-cell recording, 2-photon microscopy, and optogenetics, we explored I→I STDP at layer 2/3 (L2/3) VIP IN outputs onto L5 Martinotti cells (MCs) and basket cells (BCs). We found that VIP IN→MC synapses underwent causal long-term depression (LTD) that was presynaptically expressed. VIP IN→BC connections, however, did not undergo any detectable plasticity. Conversely, using extracellular stimulation, we explored E→I STDP at inputs to VIP INs which revealed long-term potentiation (LTP) for both causal and acausal timings. Taken together, our results demonstrate that VIP INs possess synapse-type-specific learning rules at their inputs and outputs. This suggests the possibility of harnessing VIP IN long-term plasticity to control activity-related neuropathologies such as epilepsy.

## Introduction

It has long been believed that excitatory to excitatory (E→E) long-term plasticity underlies information storage in the brain (Bliss and Collingridge, 1993; Malenka and Bear, 2004; Nabavi et al., 2014) as well as circuit remapping during development (Katz and Shatz, 1996; Cline, 1998). The brain, however, is also made up of numerous inhibitory IN types that play an active role in shaping cortical circuits through plasticity (Yazaki-Sugiyama et al., 2009; Vogels et al., 2011; D’Amour and Froemke, 2015; Udakis et al., 2020). For example, several studies have shown that plasticity at excitatory to inhibitory (E→I) and I→E connections also contribute to circuit rewiring and impact E→E neurotransmission (Maffei et al., 2006; Ormond and Woodin, 2009, 2011; Vogels et al., 2011). There is, however, a paucity of literature on the long-term plasticity at I→I synapses.

Spike-timing-dependent plasticity (STDP) is a biologically plausible experimental paradigm in which the millisecond temporal ordering of pre- and postsynaptic spikes determines whether long-term potentiation (LTP) or long-term depression (LTD) is elicited (Markram et al., 2011; Feldman, 2012; Markram et al., 2012). Presynaptic spiking occurring milliseconds before postsynaptic activity is referred to as causal because here the presynaptic spiking is causally related to postsynaptic activation, whereas the opposite temporal ordering is termed acausal (McFarlan et al., 2023). For classical STDP at E→E synapses, causal spiking elicits LTP whereas acausal spiking triggers LTD (Markram et al., 1997; Bi and Poo, 1998). Classical STDP is thus in agreement with the Hebbian postulate (Hebb, 1949) that ‘cells that fire together wire together’ (Lowel and Singer, 1992; Shatz, 1992), but has the extension that synaptic weakening arises from acausal firing, i.e., when the presynaptic cell fails to excite the postsynaptic cell (Stent, 1973; Debanne et al., 1994). This acausal LTD has important functional implications, for instance to achieve synaptic competition (Song et al., 2000; Song and Abbott, 2001).

Interestingly, inhibitory synapses do not always obey the classic Hebbian STDP rule (Feldman, 2012), neither at E→I (Lu et al., 2007) nor at I→E synapses (Holmgren and Zilberter, 2001; Woodin et al., 2003). As there are many different kinds of INs (Gouwens et al., 2020), there is thus an even larger set of synapse-type-specific forms of plasticity at E→I, I→E, and I→I connections. A relatively comprehensive collection of plasticity learning rules for a given brain region — known as a plasticitome (Sjöström, 2021) — is therefore required to understand the role of plasticity in local circuits (McFarlan et al., 2023).

Several IN types receive inhibitory inputs themselves (Artinian and Lacaille, 2018; Kullander and Topolnik, 2021). These I→I synapses have important implications for network activity because disinhibition — which can be mediated by weakening I→I connections — may increase network excitation via I→I→E connectivity motifs (McFarlan et al., 2023). Vasoactive intestinal peptide (VIP) INs, which primarily target basket cells (BCs) and Martinotti cells (MCs) (Pfeffer et al., 2013; Kepecs and Fishell, 2014; Tremblay et al., 2016), have consistently been implicated in I→I→E disinhibition (Letzkus et al., 2015; Artinian and Lacaille, 2018; Kullander and Topolnik, 2021). Though several studies have demonstrated that disinhibition plays a role in plasticity and in learning (Froemke et al., 2007; Niell and Stryker, 2010; Letzkus et al., 2011; Kaneko and Stryker, 2014; Fu et al., 2015; Adler et al., 2019), few studies have explored I→I plasticity directly (Sarihi et al., 2012).

The motor cortex function is important for the execution of voluntary movement and motor learning in the healthy brain. In recent years, VIP IN-mediated suppression of SST INs in the motor cortex has been shown to have a key role in promoting motor learning (Adler et al., 2019; Ren et al., 2022). VIP IN-mediated disinhibition has additionally been implicated in diseases like epilepsy (Cunha-Reis and Caulino-Rocha, 2020). Indeed, reduced VIP IN inhibitory drive in the mouse motor cortex had a protective effect on seizure initiation and duration (Khoshkhoo et al., 2017). These are thus concrete indications that VIP INs in motor cortex constitute a promising seizure control point.

In this phenomenological study, we explored STDP of disinhibitory motor cortex VIP INs using a combination of patch-clamp electrophysiology, 2-photon imaging, extracellular stimulation, and optogenetics. We describe STDP learning rules at both inputs to and outputs from VIP IN in the mouse motor cortex.

## Materials and methods

### Animals and ethics statement

The animal study was approved by the Montreal General Hospital Facility Animal Care Committee and adhered to the guidelines of the Canadian Council on Animal Care. To drive expression of Channelrhodopsin-2 (ChR2) and enhanced yellow fluorescent protein (EYFP) in VIP INs, we crossed homozygous *VIP*^*TM*1(*cre*)*Zjh*^/J mice (JAX strain 010908) (Taniguchi et al., 2011) with homozygous B6.Cg-*Gt(ROSA)26Sor*^*TM*32(*CAG–COP*4*H134*R/EYFP*)*Hze*^/J mice (also known as Ai32, JAX strain 024109) to obtain VIP^*Cre*/+^;Ai32^*flox*/+^ mice, henceforth referred to as VIP-ChR2 mice. Experiments were carried out in male and female postnatal day (P)21-P40 VIP-ChR2 mice. Animals were anesthetized with isoflurane and sacrificed once the hind-limb withdrawal reflex was lost.

### Acute brain slice electrophysiology

To optimize slice quality obtained from these relatively mature animals, we relied on a sucrose-based cutting solution containing (in mM) 200 sucrose, 2.5 KCl, 1.0 NH_2_PO_4_, 2.5 CaCl_2_, 1.3 MgCl_2_, 47 D-glucose and 26.2 NaHCO_3_. The solution was bubbled with 95% O_2_/5% CO_2_ for 10 min and cooled on ice to ∼4°C. Osmolality was adjusted to 338 mOsm with glucose, measured using Model 3300 or Osmo1 osmometers (Advanced Instruments Inc., Norwood, MA, USA).

After decapitation, the brain was removed and placed in ice-cold sucrose cutting solution. Coronal 300-μm-thick acute brain slices were prepared using a Campden Instruments 5000 mz-2 vibratome (Campden Instruments, Loughborough, UK) and ceramic blades (Lafayette Instrument, Lafayette, IN, USA). Brain slices were kept at ∼33°C in oxygenated artificial cerebrospinal fluid (ACSF), containing (in mM) 125 NaCl, 2.5 KCl, 1 MgCl_2_, 1.25 NaH_2_PO_4_, 2 CaCl_2_, 26 NaHCO_3_ and 25 glucose, bubbled with 95% O_2_/5% CO_2_, for ∼10 min and then allowed to cool at room temperature for at least 1 h before starting the recordings. Osmolality of the ACSF was adjusted to 338 mOsm with glucose. We carried out experiments with ACSF heated to 32–34°C with a resistive inline heater (Scientifica Ltd, Uckfield, UK), with temperature recorded and verified offline. Recordings were truncated or not used if outside this range.

An internal solution was prepared containing (in mM) 1 or 5 KCl, 115 K-Gluconate, 10 K-HEPES, 4 Mg-ATP, 0.3 Na-GTP, 10 Na_2_-Phosphocreatine and 0.1% biocytin. KOH was added to reach a pH of 7.2 to 7.4 and sucrose was added to reach the target osmolality of 310 mOsm. To visualize patched cells, 20 μM of Alexa 594 Hydrazide dye (Life Technologies, Eugene, OR, USA) was added to the internal solution. Patch pipettes were pulled using the P-1000 puller (Sutter Instruments, Novato, CA, USA). The pipette resistances varied between 4 and 7 MΩ.

We obtained whole-cell recordings using BVC-700A amplifiers (Dagan Corporation, Minneapolis, MN, USA) in current-clamp configuration. Amplified signals were low-pass filtered at 5 kHz and acquired at 40 kHz using PCI-6229 boards (NI, Austin, TX, USA). All data was acquired in Igor Pro 8 or 9 (WaveMetrics Inc., Lake Oswego, OR, USA) using custom software (Sjöström et al., 2001; Sjöström et al., 2003).^1^ We monitored input resistance, series resistance, perfusion temperature, and resting membrane potential during experiments and performed further analyses offline. We did not compensate for series resistance, nor did we account for the liquid junction potential (10 mV).

**FIGURE 1:**
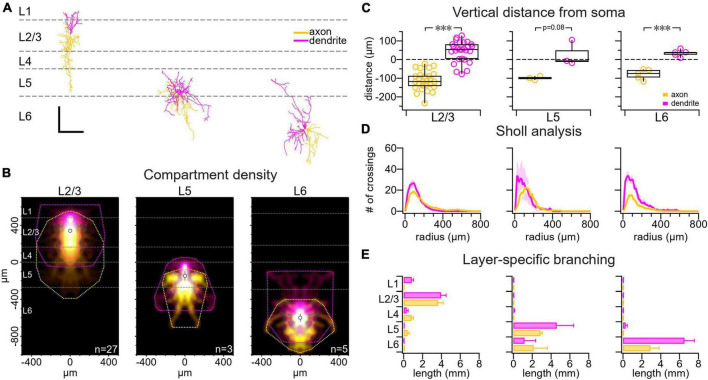
VIP IN morphology varied with cortical layer. **(A)** Sample reconstructions from a L2/3 VIP IN (left), a L5 VIP IN (middle), and a L6 VIP IN (right). Scale bar is 250 μm for both axes. Axons are labeled yellow and dendrites are labeled pink throughout the figure. **(B)** Compartment density heat map for L2/3 VIP INs (*n* = 27 cells, *N* = 21 animals), L5 VIP INs (*n* = 3 cells, *N* = 3 animals), and L6 VIP INs (*n* = 5 cells, *N* = 4 animals) appear to be vertically asymmetric. Heat maps are centered vertically on the boundary between L4 and L5. **(C)** Comparing the axonal and dendritic compartment center of mass vertically, we found that VIP IN dendrites branched upwards toward the pial surface whereas VIP IN axons chiefly projected toward deeper cortical layers (L2/3 VIP IN axon: –110 μm ± 8 μm vs. L2/3 VIP IN dendrite: 44 μm ± 10 μm, *t*-test *p* < 0.001; L5 VIP IN axon: –100 μm ± 6 μm vs. L5 VIP IN dendrite: 27 μm ± 40 μm, *t*-test *p* = 0.08; L6 VIP IN axon: –80 μm ± 10 μm vs. L6 VIP IN dendrite: 34 μm ± 8 μm, *t*-test *p* < 0.001). Dashed line: soma location. **(D)** Sholl analysis revealed that VIP IN dendrites and axons branch most densely at 100 μm from the soma. **(E)** Layer-specific branching revealed that L2/3 VIP IN axons extended into deeper cortical layers, while their dendrites were mostly localized to superficial layers. Axonal and dendritic arbors in L5 and L6 VIP INs were mostly localized to deeper cortical layers. Branching was measured as the total branch length cumulated across cells.

Cells were patched with a LUMPlanFL N 40 × /0.80 objective (Olympus, Olympus, Melville, NY, USA) using infrared video Dodt contrast on a custom-modified Scientifica SliceScope as previously described (Buchanan et al., 2012). A Chameleon ULTRA II (Coherent, Santa Clara, CA, USA) titanium-sapphire laser tuned to 920 or 820 nm was used to excite EYFP and Alexa 594 fluorophores, respectively. VIP INs were targeted based on EYFP expression visualized with 2-photon (2P) microscopy at 920 nm. L5 BCs and MCs were targeted based on their small round-shaped soma which were distinctly different from L5 pyramidal cells (PCs) which have a triangular-shaped soma and prominent apical dendrite. Cell identity was verified *post hoc* using electrophysiological and morphological properties (see below and Figure 1, Supplementary Figures 5, 7, and Supplementary Tables 1, 2). Briefly, BCs were characterized by their typical fast-spiking physiology, narrow action potential half width, and high rheobase as well as their densely branching axons and dendrites. MCs were characterized by their accommodating firing pattern and lower rheobase as well as their ascending axon and dangling dendrites (Silberberg and Markram, 2007; Buchanan et al., 2012; Sippy and Yuste, 2013; Tremblay et al., 2016).

### Long-term plasticity experiments

To explore long-term plasticity at VIP IN outputs, BCs and MCs were targeted for whole-cell recording in acute slices from P21-P40 VIP-ChR2 mice. L2/3 VIP INs were visualized using 2P microscopy at 920 nm. To activate ChR2-expressing L2/3 VIP INs, a blue laser (1-W 445-nm Blue Laser Diode Module, Item Id: 131542738201, Laserland, eBay.ca) was guided into the same light path as the 2P beam using a dichroic (FF665-Di02, Semrock Inc., Rochester, NY, USA) and controlled with a pair of 6215H 3-mm galvanometric mirrors (Cambridge Technologies, Bedford, MA, USA). The blue laser was gated by the MultiPatch software described above, thus enabling synchronization with electrophysiology acquisition. Blue laser pulses aimed onto fluorescent L2/3 VIP INs had a power of 20 mW and a duration of either 2 ms or 5 ms. L5 BCs and MCs that showed inhibitory postsynaptic potentials (IPSPs) in response to ChR2 activation were used for experiments.

To explore long-term plasticity at VIP IN inputs, L2/3 VIP INs were targeted for whole-cell recording using 2P microscopy at 920 nm. An extracellular stimulating pipette filled with ACSF was brought into the slice ∼100–200 μm from the patched cell and was used to activate VIP IN inputs. Extracellular stimulation was performed using a Biphasic Stimulation Isolator BSI-950 (Dagan Corporation, Minneapolis, MN, USA) that was manipulated via the MultiPatch software described above. Extracellular stimulation pulses were 100 μs in duration. Excitatory postsynaptic potential (EPSP) responses in patched VIP INs were inspected to ensure they were due to the activation of VIP IN inputs rather than direct stimulation of the patched VIP IN itself. A depolarization onset that emerged directly from the stimulation artifact was indicative of direct stimulation, whereas a depolarization onset that occurred 1–2 ms after the stimulation artifact was indicative of indirect stimulation. An input-output curve was used to measure the response amplitude to incremental increases in extracellular stimulation strength in the patched cell. The stimulation strength that yielded EPSPs at least 1 mV in amplitude and below the spiking threshold was used for the experiment.

For long-term plasticity experiments at VIP IN inputs and outputs, an initial pre-induction baseline consisted of two laser or extracellular stimulation pulses followed by two current pulses, both delivered at 30 Hz and offset by 700 ms, repeated 60 times over a period of 10 min. The induction protocol consisted of five laser or extracellular stimulation pulses and five current pulses delivered at 50 Hz and offset by ± 10 or +25 ms or delivered at 20 Hz and offset by ± 25 ms. The induction protocol was repeated 15 times for 2.5 min. The post-pairing baseline — which was contents-wise identical to the initial baseline — was repeated for up to 1 h. Control experiments had only presynaptic (pre only) or only postsynaptic spiking (post only) during the induction period. The time window for quantifying post-induction synaptic response amplitude started 10 min after the end of the induction and continued until the end of each individual experiment. This was compared to or normalized to the synaptic responses acquired during the pre-induction baseline period.

The paired-pulse ratio (PPR) was calculated as IPSP_2_/IPSP_1_ or EPSP_2_/EPSP_1_ for the pre-pairing and post-pairing periods. The change in PPR (ΔPPR) was calculated as PPR_after_ - PPR_before_. Coefficient of variation (CV) analysis was performed as previously described (Brock et al., 2020). Briefly, the mean and CV of IPSP_1_ or EPSP_1_ were calculated for the pre-pairing period, followed by normalizing mean and CV^–2^ to the post-pairing period. The angle (θ) was defined by the diagonal unity line and the line formed by linking the starting coordinate (1, 1) and CV analysis endpoint. θ < 0 (i.e., clockwise from diagonal) indicated a postsynaptic locus of plasticity expression, while θ > 0 indicated a presynaptic locus (Brock et al., 2020).

### Identification of motor cortex layers

The motor cortex was targeted based on the location of the corpus callosum white matter tract. L1 and white matter were identified based on their lack of cell bodies. For electrophysiological experiments, we differentiated between L2/3, L5 and, L6 based on PC morphology. In L2/3, PC somata are relatively small, whereas in L5, PCs have large somata and a thick apical dendrite. L6 PCs have rounded somata and a thin apical dendrite.

Layer boundaries for immunohistochemistry and biocytin histology were informed by NeuN cell counts and *in situ* hybridization (ISH) data from the Allen Institute Mouse Brain Atlas (Lein et al., 2007). We selected ISH images stained for Stard8, Rorb, Bend5, and Ighm expression, which was restricted to L2/3, L4, L5, and L6, respectively. Using Fiji/ImageJ (Schindelin et al., 2012), we selected a ∼800-μm-wide linear region of interest spanning the motor cortex from pial surface to white matter and measured the intensity profile across the cortical thickness. We thus overlayed the intensity profile for each gene marker (Supplementary Figure 1). Each density profile was baseline-subtracted, peak-normalized, and box-smoothed (setting 10) in Igor Pro 9. The point of intersection between pixel intensity profiles were then used to define layer boundaries. The percentage distance normalized from pial surface to white matter was used to inform layer boundaries in individual slices.

We were concerned that fixed tissue samples might be altered, e.g., due to fixation, cover slipping, or other histology steps, which might distort the apparent cortical thickness. We therefore verified the cortical thickness in fixed tissue by comparing to the acute slice in electrophysiology experiments, which revealed a good match, thus validating the accuracy of fixed slice samples.

### Immunohistochemistry

P21-P40 VIP-ChR2 mice were anesthetized with isofluorane and transcardially perfused with 0.1 M phosphate buffered saline (PBS) followed by 4% paraformaldehyde. Brains were incubated in 4% paraformaldehyde overnight, and then stored for two additional days in a 30% (w/v) sucrose solution. Next, brains were mounted in plastic cubes containing Optimal Cutting Temperature embedding medium (25608-930, VWR, Radnor, PA, USA) and then frozen using a bath of 100% EtOH and dry ice. Fixed brains were sectioned with a cryostat at 50 μm thickness through the motor cortex and sections were placed in a 0.01 M PBS solution. Sections underwent a 20-min wash in 0.01 M PBS with 1% Triton-X followed by a 90-min wash in 0.01 M PBS with 0.3% Triton-X and 10% normal donkey serum (NDS; 017-000-121 Jackson ImmunoResearch, West Grove, PA, USA). All antibody incubations were performed in 0.01 M PBS with 0.3% Triton-X and 1% NDS.

Sections were incubated overnight at 4°C in the following primary antibodies: 1:500 rabbit anti-VIP (20077, ImmunoStar, Dietzenbach, Germany), 1:100 rat anti-somatostatin (ab30788, Abcam, Boston, MA, USA), 1:1000 chicken anti-GFP (ab13970, Abcam), 1:500 mouse anti-parvalbumin (p3088, Sigma, St. Louis, MO, USA), and 1:1000 mouse anti-NeuN (ab104224, Abcam). Twenty-four hours later, tissue underwent three 15-min washes in 0.01 M PBS with 0.3% Triton-X and 1% NDS, followed by a 90-min incubation in the following Alexa Fluor secondary antibodies at 1:1000: donkey anti-rabbit 647 (711-605-152, Jackson ImmunoResearch), donkey anti-rat 594 (712-585-150, Jackson ImmunoResearch), donkey anti-chicken 488 (703-545-155, Jackson ImmunoResearch), goat anti-mouse 647 (A21240, ThermoFisher Scientific, Waltham, MA, USA), and donkey anti-mouse 568 (SAB4600075, Sigma). Next, the tissue underwent three 20-min washes in 0.01 M PBS with 0.3% Triton-X and 1% NDS. Following this procedure, coronal slices were mounted using coverslips with a 40 μl bolus of ProLong Gold Antifade Mountant (ThermoFisher Scientific).

Sections were imaged using a Fluoview FV1000 confocal laser scanning microscope and Fluoview software (Olympus Canada, Richmond Hill, ON, Canada) or a Zeiss LSM780 confocal laser scanning microscope and ZEN software (Zeiss, Oberkochen, Germany). Image analysis and quantification were performed using Fiji/ImageJ (Schindelin et al., 2012) and Igor Pro 9 (Wavemetrics). Cell counts for neurons expressing VIP, EYFP, somatostatin (SST) and parvalbumin (PV) were carried out across all six cortical layers and in both hemispheres.

### Biocytin histology and morphological reconstructions

Patched VIP INs, MCs, and BCs used in long-term plasticity experiments were saved for neuronal reconstruction. Once the experiment was completed, the patch pipette was removed slowly while applying light positive pressure. Sections were then incubated in 4% paraformaldehyde overnight and were stored in 0.01 M PBS solution for up to 3 weeks before staining.

Sections underwent four 10-min washes in 0.01 M Tris-buffered saline (TBS) solution with 0.3% Triton-X followed by a 1-h wash in 0.01 M TBS with 0.3% Triton-X and 10% NDS. Sections were incubated overnight at 4°C in 0.01 M TBS with 0.3% Triton-X and 1% NDS, supplemented with 1:200 Alexa Fluor 647- or Alexa fluor 488-conjugated Streptavidin (ThermoFisher Scientific). Twenty-four hours later, tissue underwent four 10-min washes in 0.01 M TBS. Following this procedure, sections were mounted using coverslips with a 40 μl bolus of ProLong Gold Antifade Mountant (ThermoFisher Scientific). 3D image stacks were acquired using a Zeiss LSM780 confocal laser scanning microscope and ZEN software (Zeiss) and used for morphological reconstructions.

3D confocal image stacks were contrast adjusted and converted to 8 bits or 16 bits in Fiji (Schindelin et al., 2012) and then imported into Neuromantic V1.7.5 (Myatt et al., 2012) for manual tracing. Morphometry was performed in Igor Pro 9 (Wavemetrics) using the qMorph in-house custom software as previously described (Buchanan et al., 2012; Zhou et al., 2021).^2^

### Statistics

Unless otherwise noted, results are reported as the mean ± standard error of the mean (SEM). Significance levels are denoted using asterisks (*p < 0.05, **p < 0.01, ***p < 0.001). Pairwise comparisons were carried out using a two-tailed Student’s *t*-test for equal means. If an equality of variances *F* test gave *p* < 0.05, we employed the unequal variances *t*-test. Wilcoxon-Mann-Whitney’s non-parametric test was always used in parallel to the *t*-test, yielding similar outcomes. Statistical tests were performed in Igor Pro 9 (Wavemetrics).

## Results

### The VIP-ChR2 mouse line reliably identifies VIP INs

We created a VIP-ChR2 mouse line by crossing a VIP-Cre driver line with the Ai32 ChR2/EYFP reporter line (Methods). We validated our VIP-ChR2 mice by exploring the degree of overlap between the genetic EYFP tag and VIP expression. To do so, we relied on immunohistochemistry (Methods). This revealed that ∼80% of EYFP-positive cells were also positive for VIP and that ∼88% of VIP-positive cells were also positive for EYFP (Supplementary Figure 2), demonstrating that our VIP-ChR2 mouse line was highly specific for VIP INs, in agreement with the prior literature (Taniguchi et al., 2011).

Next, we looked at the spatial distribution of VIP INs across the cortical layers. Similar to what has been shown in the barrel cortex (Prönneke et al., 2015; Almási et al., 2019) and visual cortex (Gonchar et al., 2007), we found in the motor cortex that most VIP INs were located in L2/3 (Supplementary Figure 3). We also explored whether VIP INs expressed SST or PV — molecular markers of MCs and BCs, respectively (Blackman et al., 2013) — but found that they did not (Supplementary Figure 4), in agreement with the prior literature (Prönneke et al., 2015).

### L2/3, L5, and L6 VIP INs are electrophysiologically indistinguishable

We compared the electrophysiological properties of a total of 46 patched VIP INs from L2/3, L5, and L6. We found no detectable differences across layers in basic electrophysiological properties such as resting membrane potential, firing threshold, action potential height, action potential half width, rheobase, membrane time constant, and input resistance (Supplementary Table 1). We also found that VIP INs had varying spike patterns. Following the Petilla convention (Ascoli et al., 2008), we found that VIP INs exhibited three different action potential firing patterns: adapting, burst firing, and irregular firing (Supplementary Figure 5 and Supplementary Table 1).

### L2/3, L5, and L6 VIP INs morphologies vary with cortical layer

We investigated the morphology of VIP INs in the mouse motor cortex. We used biocytin histology and confocal imaging to 3D reconstruct patched VIP INs in L2/3, L5 and L6. Consistent with previous findings in the barrel cortex (Prönneke et al., 2015), we found that VIP INs have dendrites that project toward the pial surface and axons that extend into deeper cortical layers (Figures 1A–C). Sholl analysis (Sholl, 1953) additionally revealed that VIP IN dendrites and axons were most densely branched around 100 μm away from the soma (Figure 1D). L2/3 VIP IN dendrites were mostly localized to superficial layers, whereas their axons extended into deeper cortical layers. L5 and L6 VIP INs dendrites and axons were mostly localized to deeper cortical layers (Figure 1E).

### Long-term plasticity at VIP IN outputs

#### Reliable optogenetic activation of VIP INs

Because L2/3 VIP INs are mostly found in L2/3 (Supplementary Figure 3; Gonchar et al., 2007; Prönneke et al., 2015; Almási et al., 2019) and because L2/3 VIP INs are more homogeneous compared to other layers (Gouwens et al., 2020), we decided to target these INs for the subsequent experiments exploring long-term plasticity at VIP IN inputs and outputs. We first targeted L2/3 VIP INs for whole-cell recording and used blue laser light to explore whether we could reliably drive ChR2 to spike the cell. We found that blue laser light reliably evoked spiking up to 50 Hz (Supplementary Figure 6).

#### VIP IN→MC connections exhibited causal LTD

**FIGURE 2:**
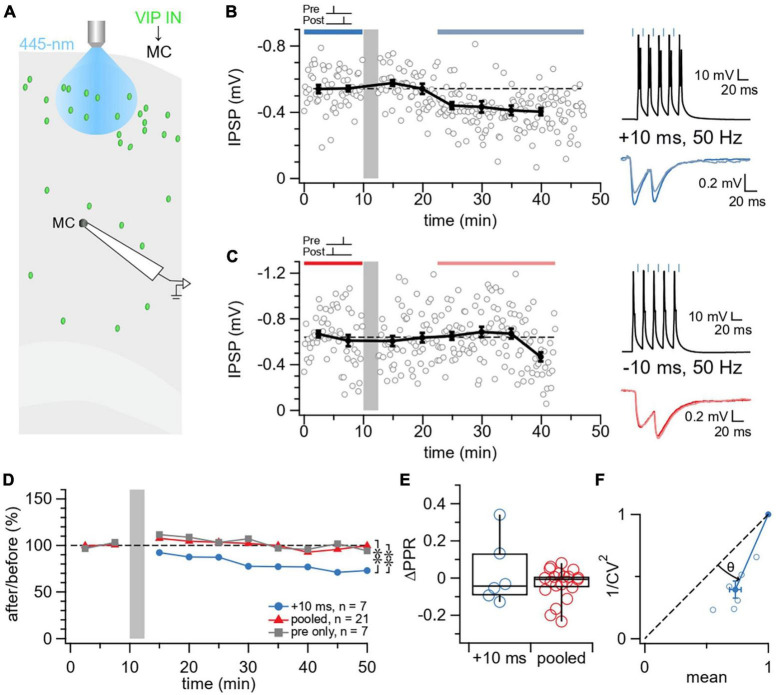
Causal STDP yielded LTD at VIP IN→MC synapses. **(A)** Schematic illustrating the patched L5 MC and laser-activated L2/3 VIP INs in the motor cortex. **(B)** Sample experiment from a whole-cell recording at a VIP IN→MC connection using an induction paradigm (gray bar) of 50 Hz, +10 ms revealed how LTD was elicited in the postsynaptic MC (after/before = 75%, *p* < 0.001). The dark blue bar (pre-pairing) and light blue bar (post-pairing) indicate the time window for plasticity quantification. Causal pre-pairing IPSPs are labeled in dark blue, causal post-pairing IPSPs are labeled in light blue. **(C)** Sample experiment from a whole-cell recording at a VIP IN→MC connection using an induction paradigm (gray bar) of 50 Hz, –10 ms showed that no change in IPSP response was elicited in the postsynaptic MC (after/before = 97%, *p* = 0.59). The red bar (pre-pairing) and pink bar (post-pairing) indicate the time window for plasticity quantification. Acausal pre-pairing IPSPs are labeled in red, acausal post-pairing IPSPs are labeled in pink. **(D)** Ensemble averages showed that causal LTD only occurs at VIP IN→MC synapses with an induction paradigm of 50 Hz, +10 ms (ANOVA *p* < 0.05; +10 ms: 78% ± 6%, *n* = 7 connections, *N* = 7 animals, vs. pooled: 98% ± 4%, *n* = 21 connections, *N* = 18 animals, *t*-test *p* < 0.01; +10 ms vs. pre only controls: 100% ± 4%, *n* = 7 connections, *N* = 7 animals, *t*-test *p* < 0.01; pooled vs. pre only controls, *t*-test *p* = 0.68). **(E)** VIP IN→MC LTD did not change ΔPPR compared to the pooled group (+10 ms: 0.027 ± 0.07 vs. pooled: –0.032 ± 0.02, Wilcoxon test *p* = 0.98), suggesting a postsynaptic locus of expression for plasticity. **(F)** For CV analysis, points below diagonal for VIP IN→MC LTD (Wilcoxon test, θ = 22° ± 2°, *p* < 0.01) suggests that IPSP suppression was due to a reduction in presynaptic release (Brock et al., 2020).

In acute slices from VIP-ChR2 mice, we targeted MCs for whole-cell recording and optogenetically activated presynaptic VIP INs to explore how plasticity of VIP IN→MC synapses depend on spike rate and timing (Figure 2A). We found that MC disinhibition was possible by inducing LTD at 50 Hz firing rate and causal timing difference of Δt = +10 ms (Figure 2B). In contrast, we found no plasticity at other tested timings and frequencies (pooled: 50 Hz and Δt = −10 ms, +25 ms; 20 Hz and Δt = ±25 ms) or in control experiments with no postsynaptic spiking (Figures 2C, D).

We then assessed the locus of expression of VIP IN→MC LTD. We utilized two independent methods for determining the pre- versus postsynaptic locus, synaptic response PPR (Blackman et al., 2013) and CV analysis (Brock et al., 2020). We found that VIP IN→MC LTD did not change ΔPPR compared to controls, suggesting a postsynaptic locus of expression (Figure 2E). In contrast with ΔPPR, VIP IN→MC LTD reduced 1/CV^2^ (Figure 2F), suggesting decreased presynaptic release (Blackman et al., 2013; Brock et al., 2020).

#### No STDP detected at VIP IN→BC connections

**FIGURE 3:**
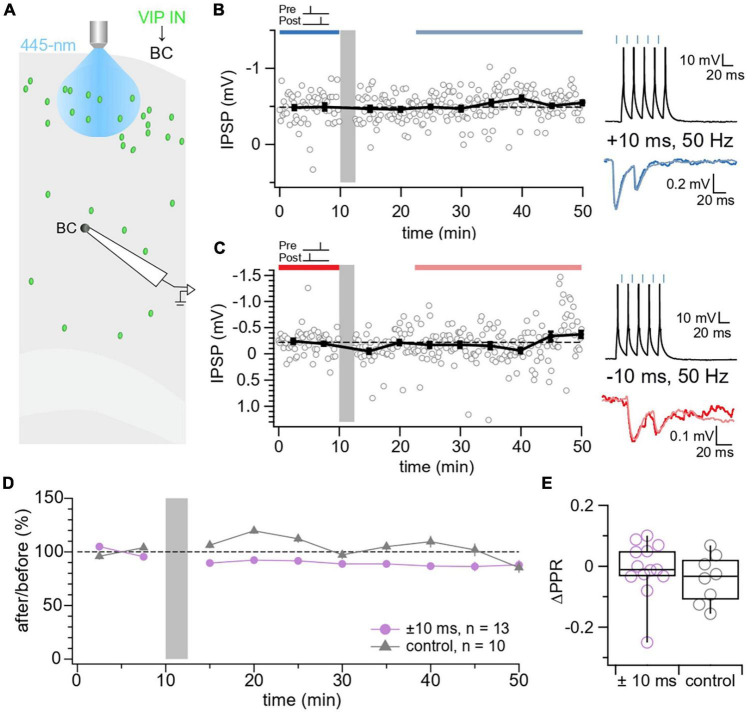
No STDP detected at VIP IN→BC synapses. **(A)** Schematic illustrating the patched L5 BC and laser-activated L2/3 VIP INs in the motor cortex. **(B)** Sample experiment from a whole-cell recording at a VIP IN→BC connection using an induction paradigm (gray bar) of 50 Hz, +10 ms revealed that no change in IPSP response was elicited in the postsynaptic BC (after/before = 106%, *p* = 0.27). The dark blue bar (pre-pairing) and light blue bar (post-pairing) indicate the time window for plasticity quantification. Causal pre-pairing IPSPs are labeled in dark blue, causal post-pairing IPSPs are labeled in light blue. **(C)** Sample experiment from a whole-cell recording at a VIP IN→BC connection using an induction paradigm (gray bar) of 50 Hz, –10 ms showed that no change in IPSP response was elicited in the postsynaptic BC (after/before = 93%, *p* = 0.75). The red bar (pre-pairing) and pink bar (post-pairing) indicate the time window for plasticity quantification. Acausal pre-pairing IPSPs are labeled in red, acausal post-pairing IPSPs are labeled in pink. **(D)** Ensemble averages showed that VIP IN→BC synapses did not undergo any detectable plasticity following our induction protocol ( ± 10 ms: 89% ± 6%, *n* = 13 connections, *N* = 13 animals vs. control: 102% ± 8%, *n* = 10 connections, *N* = 9 animals, *t*-test *p* = 0.22). Pre only (*n* = 5 connections, *N* = 4 animals) and post only (*n* = 5 connections, *N* = 5 animals) control conditions were indistinguishable (*t*-test *p* = 0.89) and were therefore pooled in one control group. **(E)** There was no change in ΔPPR at VIP IN→BC synapses ( ± 10 ms: –0.0075 ± 0.03 vs. control: –0.041 ± 0.03, *t*-test *p* = 0.35).

We next explored STDP at VIP IN→BC synapses. In our VIP-ChR2 mice, we targeted motor cortex L5 BCs for whole-cell recording and selectively activated L2/3 VIP INs using blue laser light (Figure 3A). Unlike VIP IN→MC synapses, we found that VIP IN→BC synapses exhibited no detectable STDP (Figures 3B–E).

#### Post-hoc identification of MCs and BCs

MCs and BCs were identified based on their distinct electrophysiological and morphological properties (Tremblay et al., 2016). Compared to fast-spiking BCs, MCs had an adapting firing pattern with a lower rheobase, higher input resistance, larger spike half width, and longer membrane time constant (Supplementary Table 2). MCs had a single characteristically ascending axon and dangling dendrites, while BCs had highly locally branching axonal and dendritic arbors that were relatively radially symmetric (Supplementary Figure 7).

### Long-term plasticity at VIP inputs

#### E→VIP IN connections exhibit LTP irrespective of temporal order

**FIGURE 4:**
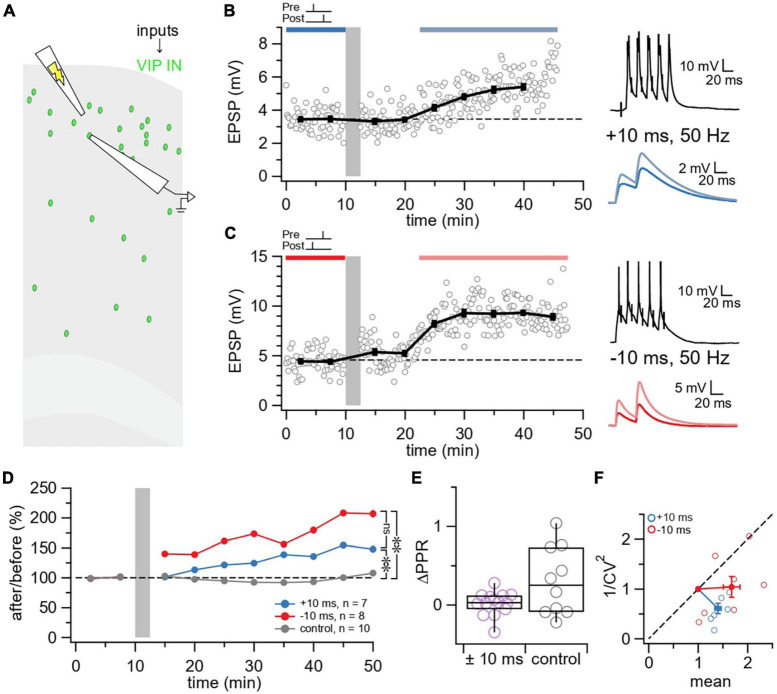
VIP inputs exhibit LTP irrespective of temporal order. **(A)** Schematic illustrating the activation of L2/3 VIP IN inputs using extracellular stimulation in the motor cortex. **(B)** Sample experiment from a whole-cell recording at a L2/3 VIP IN using an induction paradigm (gray bar) of 50 Hz, +10 ms revealed that LTP was elicited at this E→VIP IN synapse (after/before = 147%, *p* < 0.001). The dark blue bar (pre-pairing) and light blue bar (post-pairing) indicate the time window for plasticity quantification. Causal pre-pairing EPSPs are labeled in dark blue, causal post-pairing EPSPs are labeled in light blue. **(C)** Sample experiment from a whole-cell recording of a L2/3 VIP IN using an induction paradigm (gray bar) of 50 Hz, –10 ms revealed that LTP was elicited at this E→VIP IN synapse (after/before = 203%, *p* < 0.001). The red bar (pre-pairing) and pink bar (post-pairing) indicate the time window for plasticity quantification. Acausal pre-pairing EPSPs are labeled in red, acausal post-pairing EPSPs are labeled in pink. **(D)** Ensemble averages showed that LTP is induced at E→VIP IN synapses at 50 Hz, ± 10 ms (Brown-Forsythe ANOVA *p* < 0.01; +10 ms: 140% ± 6%, *n* = 7 connections, *N* = 4 animals vs. control: 99% ± 9%, *n* = 10 connections, *N* = 8 animals, Wilcoxon-Mann-Whitney test *p* < 0.01; –10 ms: 168% ± 17%, *n* = 8 connections, *N* = 7 animals vs. control, Wilcoxon-Mann-Whitney test *p* < 0.01; +10 ms vs. –10 ms, Wilcoxon-Mann-Whitney test *p* = 0.23). Pre only (*n* = 5 connections, *N* = 5 animals) and post only (*n* = 5 connections, *N* = 3 animals) control conditions were indistinguishable (Wilcoxon-Mann-Whitney test *p* = 0.15) and were therefore pooled in one control group. **(E)** There was no change in ΔPPR following E→VIP IN LTP ( ± 10 ms: 0.019 ± 0.04, *n* = 14 connections, *N* = 10 animals vs. control: 0.30 ± 0.1, *n* = 10 connections, *N* = 8 animals, Wilcoxon test *p* = 0.17). **(F)** For CV analysis, points below diagonal for E→VIP IN LTP (+10 ms: Wilcoxon test, θ = 266° ± 9°, *p* < 0.001; –10 ms: Wilcoxon test, θ = 234° ± 19°, *p* < 0.001) indicated that plasticity was postsynaptically expressed (Brock et al., 2020).

Next, we studied long-term plasticity at VIP IN inputs. We patched L2/3 VIP INs and used extracellular stimulation to readily recruit excitatory inputs onto VIP INs (Figure 4A). We found that E→VIP IN synapses were potentiated at 50 Hz firing rate with both causal and acausal timings of Δt = ±10 ms (Figures 4B–D).

We then assessed whether E→VIP IN LTP was expressed presynaptically or postsynaptically. We found that E→VIP IN LTP did not change PPR (Figure 4E), in keeping with postsynaptically expressed LTP. In agreement, E→VIP IN LTP did not reduce 1/CV^2^ (Figure 4F), which also suggested a postsynaptic locus of expression (Blackman et al., 2013; Brock et al., 2020).

## Discussion

**FIGURE 5:**
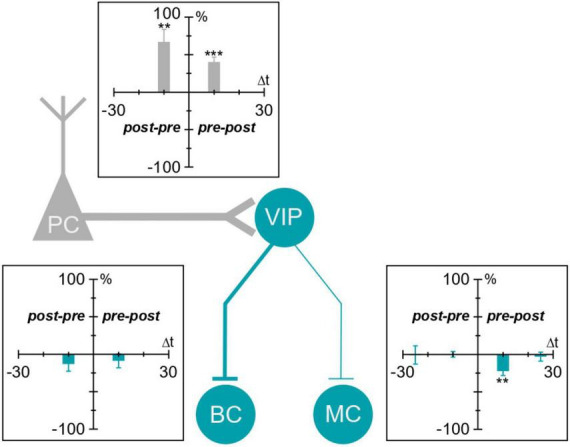
Plasticity of VIP IN inputs and outputs in the motor cortex. VIP IN inputs and outputs in the motor cortex undergo plasticity. We found that VIP IN inputs (labeled in gray) were potentiated with both causal and acausal timings. VIP IN outputs (labeled in blue) underwent causal LTD at VIP IN→MC synapses. VIP IN→BC synapses, on the other hand, did not exhibit any detectable plasticity. **p < 0.01, ***p < 0.001.

VIP IN-mediated disinhibition has consistently been shown to boost learning and plasticity in cortical circuits (Fu et al., 2014; Fu et al., 2015; Adler et al., 2019). Yet, to our knowledge, no studies have explored the plasticity of VIP INs themselves. Here, we described the phenomenology of long-term plasticity at VIP IN inputs and outputs in the mouse motor cortex (Figure 5). We found that VIP IN→MC synapses underwent causal LTD, but we could not detect any STDP at VIP IN→BC synapses. On the input side, we found that E→VIP IN synapses potentiated for both causal and acausal timings. Taken together, our findings reveal that plasticity at VIP IN inputs and outputs is specific to synapse type.

### Synapse-type specificity

Synapse-type-specific plasticity has been reported throughout the brain (Larsen and Sjöström, 2015) and allows synapses to adapt differentially depending on factors such as target cell (Blackman et al., 2013) or functional role in the microcircuit (McFarlan et al., 2023). In our study, we found that plasticity outcomes at VIP IN outputs depend on synapse type. This synapse-type-specific plasticity has been reported at I→I synapses in L2/3 visual cortex, where presynaptic tetanic stimulation induced I→I LTP at PV IN→PV IN synapses but not at SST IN→PV IN synapses (Sarihi et al., 2012). Similarly, increased PC activity in L2/3 prefrontal cortex selectively potentiated SST IN→PC synapses but not PV IN→PC synapses (Chiu et al., 2018). Additionally, I→E synapses in the developing cortex exhibited different plasticity rules depending on the location of the postsynaptic cell. For example, auditory cortex L4 IN→L5 PC synapses exhibited causal LTP (Field et al., 2020), whereas visual cortex L4 BC→L4 PC synapses exhibited causal LTD that later switched to LTP after critical period sensory experience (Vickers et al., 2018). The switch in plasticity rules at L4 BC→L4 PC synapses suggested that these I→E synapses may be important for promoting plasticity, whereas L4 IN→L5 PC synapses may provide stability. Overall, these studies highlight the many factors that contribute to synapse-type-specific plasticity which include cell type, cell location, and synapse type.

In our study, plasticity was induced at VIP IN→MC synapses but not VIP IN→BC synapses. Considering that VIP INs weakly synapse with BCs compared to MCs (Pfeffer et al., 2013; Kepecs and Fishell, 2014; Tremblay et al., 2016; Apicella and Marchionni, 2022; Campagnola et al., 2022), it is possible that VIP INs and BCs do not have enough consistent coincident activity to induce STDP at VIP IN→BC synapses. Induction of plasticity at VIP IN→BC synapses may instead depend on pre- or postsynaptic activity alone. High frequency stimulation of PV INs, for example, resulted in E→I LTP and I→I LTP in the visual cortex (Sarihi et al., 2012; Chistiakova et al., 2019). Moreover, low frequency theta-burst stimulation at excitatory inputs onto hippocampal BCs resulted in E→I LTP, whereas high frequency theta-burst stimulation resulted in E→I LTD (Camiré and Topolnik, 2014). Other factors such as neuromodulators may be required for VIP IN→BC plasticity. It was demonstrated that the release of the neuropeptide enkephalin by hippocampal VIP INs long-term disinhibited CA2 PCs via I→E LTD at PV IN outputs. This VIP IN-mediated disinhibition allowed for enhanced information transfer between hippocampal CA3 and CA2 PCs, which was important for social memory storage (Leroy et al., 2022). Thus, this form of plasticity did not require the coincident firing of pre and postsynaptic cells, but rather a neuromodulator. Together, these results highlight the need to further explore different induction protocols to elucidate the plasticity rules that govern VIP IN→BC synapses.

### VIP IN plasticity depends on spike timing

Though plasticity at a given synapse type depends on several factors including rate, timing, depolarization, and higher-order spiking statistics (Sjöström et al., 2001; Froemke and Dan, 2002; Froemke et al., 2006; Pfister and Gerstner, 2006), we focused here on how plasticity at VIP IN inputs and outputs depends on the relative timing of pre- and postsynaptic spiking. We found that LTD induction at VIP IN→MC synapses was sensitive to timing (Figure 2). At E→VIP IN synapses, however, LTP did not depend on the sign of relative timing, yet pre- or postsynaptic firing alone yielded no plasticity (Figure 4). This demonstrated that this form of LTP was still dependent on spike timing, a defining feature of STDP (Markram et al., 2012). Previous studies have additionally shown that STDP can be symmetric around the origin (Egger et al., 1999; Abbott and Nelson, 2000; Lu et al., 2007). Taken together, our findings highlight how timing is a key determinant of VIP IN STDP.

### The locus of expression

The locus of expression of long-term plasticity is important because it carries with it computational implications (Costa et al., 2015; Costa et al., 2017; Mizusaki et al., 2022). Postsynaptically expressed plasticity generally alters only synaptic strength while leaving short-term dynamics unaffected, although there are exceptions to this rule (Poncer and Malinow, 2001). Presynaptically expressed long-term plasticity, however, typically alters both short-term plasticity and synaptic gain (Markram and Tsodyks, 1996; Sjöström et al., 2003; Sjöström et al., 2007). This is because during high-frequency spike trains, the readily releasable pool of vesicles is depleted, which causes short-term synaptic depression (Zucker and Regehr, 2002), although at some synapse, short-term facilitation dominates for other mechanistic reasons (Blackman et al., 2013). Either way, these short-term synaptic dynamics are generally strongly affected by pre- but not by postsynaptically expressed long-term plasticity. Short-term plasticity is functionally important as it filters information transferred by a synapse (Fortune and Rose, 2001; Fuhrmann et al., 2002). In this view, short-term facilitating connections act as high-pass filtering burst detectors (Maass and Zador, 1999; Matveev and Wang, 2000), while short-term depressing connections low-pass filter information for correlation detection and gain-control (Abbott et al., 1997; Rosenbaum et al., 2012). For instance, presynaptic LTP would be expected to increase the release probability, thereby depleting the readily-releasable pool of vesicles faster during high-frequency bursts. The ensuing increase in short-term depression thus biases the synapse toward correlation detection at the expense of burst detection (Blackman et al., 2013; Costa et al., 2017).

In our study, we analyzed PPR and CV to assess the locus of expression. For VIP IN→MC LTD, we found no change in PPR, suggesting a postsynaptic locus of expression (Figure 2E), whereas CV analysis suggested that plasticity was expressed presynaptically (Figure 2F; Brock et al., 2020). This apparent discrepancy could be explained by the paired-pulse stimulation frequency not being potent enough to sufficiently deplete the readily-releasable pool of vesicles, leading it to be inconclusive. With this interpretation, LTD at VIP IN→MC was presynaptically expressed. In this view, this form of LTD would thus be expected to alter the information filtering of VIP IN→MC synapses in addition to weakening them (Blackman et al., 2013; Costa et al., 2017).

Mechanistically, presynaptically expressed plasticity at VIP IN→MC synapses could be mediated by retrograde messengers like endocannabinoids (Castillo et al., 2012; Piette et al., 2020) or BDNF (Inagaki et al., 2008; Vickers et al., 2018). Additionally, given that the postsynaptic MC needs to be active in order to coincidentally fire with the presynaptic VIP IN, postsynaptic NMDA receptor signaling may be involved. Indeed, NMDA receptor signaling has been shown to control GABA_A_ receptor stability at inhibitory synapses (Muir et al., 2010).

For inputs to VIP INs, on the other hand, ΔPPR and CV analysis agreed that E→VIP IN LTP had a postsynaptic locus of expression (Figure 4). The expression of postsynaptic E→E LTP is typically due to the insertion of postsynaptic AMPA receptors (Malinow and Malenka, 2002; Bredt and Nicoll, 2003; Kessels and Malinow, 2009). The NMDA receptor, which is particularly well suited for STDP given its capacity for coincidence detection, is also highly implicated in E→E LTP (Wong et al., 2021). Thus, AMPA receptor insertion and NMDA receptor signaling are strong candidates for the postsynaptic expression of E→I LTP at VIP IN inputs.

### The consequences of VIP IN plasticity

To understand the consequences of long-term plasticity at VIP IN inputs and outputs, it is important to consider its impact at the circuit level (McFarlan et al., 2023). LTP of excitatory inputs to VIP INs promotes VIP IN activity, which by inhibiting BCs and MCs is expected to increase circuit activity overall. Consequently, it has been proposed that attention operates through cortical disinhibitory circuits, e.g., by neuromodulation or by top-down control (Kullander and Topolnik, 2021; Speed and Haider, 2021). It has been argued that cholinergic modulation specifically of VIP INs is required for attention (Obermayer et al., 2019), although some disagree and propose that VIP IN activation is orthogonal to attention (Myers-Joseph et al., 2023). Regardless, we show here how activity-driven VIP IN plasticity can potentially contribute to such attentional effects.

The plasticity of VIP INs may also more generally regulate learning. For instance, VIP IN-mediated suppression of SST INs has been correlated with improved motor learning in the motor cortex (Adler et al., 2019) as well as enhanced adult plasticity in the visual cortex (Fu et al., 2014; Fu et al., 2015). Thus, E→I LTP at VIP IN inputs may help to boost learning and plasticity in cortical circuits.

In addition, VIP IN→MC LTD is expected to increase inhibition specifically of PC dendrites (Wang et al., 2004). Although E→VIP IN LTP and VIP IN→MC LTD may superficially seem to oppose each other if triggered simultaneously, they would additionally redistribute inhibition across the somato-dendritic axis of pyramidal cells (Pouille and Scanziani, 2004; Blackman et al., 2013). According to influential theoretical frameworks on cortical associative learning (Larkum, 2013), such dendritic inhibition is expected to regulate neocortical information storage.

As VIP INs are able to mediate disinhibition (Artinian and Lacaille, 2018; Kullander and Topolnik, 2021), they are ideally positioned as key regulators of activity in local circuits, with implications for disease states such as epilepsy (Cunha-Reis and Caulino-Rocha, 2020). For example, it has been reported that, whereas inhibition of SST INs prolongs seizures, inhibition of VIP INs reduces seizure propensity (Khoshkhoo et al., 2017). Consequently, the protective role of VIP IN→MC LTD might be possible to harness as a therapy for epilepsy.

### Caveats

One caveat in our study is the discrepancy between ΔPPR and CV analysis for determining the locus of expression of VIP IN→MC LTD. CV analysis suggested that LTD was expressed presynaptically, whereas PPR analysis suggested it was expressed postsynaptically. This discrepancy between CV and PPR analyses could occur if the 30-Hz stimulation was not sufficiently high to deplete the readily releasable pool. It is possible that depleting VIP IN→MC synapses better would reveal a change in PPR.

Another potential caveat with our experimental paradigm comes from the use of ChR2 to activate presynaptic VIP INs. Since ChR2 fluxes calcium (Nagel et al., 2003), it may directly trigger release at synaptic boutons. ChR2 may also depolarize synaptic terminals, again contributing to release. This artifact, if present, would be expected to elevate release and increase short-term depression. Consequently, this may be particularly important when using PPR as a measure for determining the locus of plasticity expression at VIP IN outputs, whereas CV analysis may be less affected. However, given the many hundreds of micrometer distance between presynaptic laser stimulation and postsynaptic cells where VIP IN synapses form (Figures 2A, 3A), this artifact seemed unlikely.

Furthermore, the lack of single-cell resolution with respect to presynaptic cell activation — we likely optogenetically stimulated more than one presynaptic L2/3 VIP IN at a time. An alternative approach for unitary synapse resolution would be paired recordings (Lalanne et al., 2016). However, given appreciable distance between L2/3 and L5, the sparsity of VIP INs in the cortex (Supplementary Figure 3), and the overall low connectivity of VIP IN outputs (Walker et al., 2016), paired recordings would be an impractical tool. Another alternative approach for unitary synapse resolution is by using 2P optogenetic activation (Chou et al., 2023), which in the future should enable the study of long-term plasticity at multiple VIP IN→MC/BC synapses in parallel.

### Future directions

To further our understanding of how disinhibitory plasticity impacts the healthy brain as well as neuropathologies, future research will need to explore the plasticity at the many different I→I and I→I→E synapses, to contribute to the plasticitome of cortical INs (McFarlan et al., 2023). Because it is challenging to explore many different synapse types, this effort will likely require new high-throughput plasticity-mapping approaches (Sjöström, 2021).

We relied here on the STDP experimental paradigm, which is widely believed to be biologically plausible (Markram et al., 2011; Feldman, 2012; Markram et al., 2012). However, some have disagreed and instead argued that STDP is of limited biological relevance (Lisman and Spruston, 2005; Lisman and Spruston, 2010). On a related note, due to the experimental challenges associated with targeting these specific synapse types, we could only explore a limited plasticity induction parameter space in this study. Induction protocols that e.g., rely on local dendritic spikes (Holthoff et al., 2004; Remy and Spruston, 2007; Bittner et al., 2017) may thus reveal plasticity at VIP IN→BC synapses. We also did not explore neuromodulation, which may additionally gate plasticity (Pawlak et al., 2010), even synapse specifically for the same cell type (Edelmann et al., 2017). Our study is thus not a final verdict on VIP IN plasticity, but rather a starting point.

## Data availability statement

The raw data supporting the conclusions of this article will be made available by the authors, without undue reservation.

## Ethics statement

The animal study was approved by the Montreal General Hospital Facility Animal Care Committee. The study was conducted in accordance with the local legislation and institutional requirements.

## Author contributions

ARM: Conceptualization, Data curation, Formal analysis, Funding acquisition, Investigation, Methodology, Resources, Software, Visualization, Writing – original draft, Writing – review and editing, Project administration, Supervision, Validation. CG: Formal analysis, Funding acquisition, Investigation, Writing – review and editing, Data curation, Methodology. IG: Formal analysis, Investigation, Writing – review and editing, Data curation, Methodology. CW: Formal analysis, Investigation, Writing – review and editing, Data curation, Methodology. TAL: Formal analysis, Funding acquisition, Investigation, Writing – review and editing, Data curation, Methodology. PJS: Conceptualization, Data curation, Formal analysis, Funding acquisition, Investigation, Methodology, Resources, Software, Supervision, Validation, Visualization, Writing – original draft, Writing – review and editing.

## Footnotes

1
https://github.com/pj-sjostrom/MultiPatch.git

2
https://github.com/pj-sjostrom/qMorph

## References

[B1] Abbott L. F. Nelson S. B. (2000). Synaptic plasticity: Taming the beast. *Nat. Neurosci.* 3(Suppl.), 1178–1183. 10.1038/81453 11127835

[B2] Abbott L. F. Varela J. A. Sen K. Nelson S. B. (1997). Synaptic depression and cortical gain control. *Science* 275 220–224. 8985017 10.1126/science.275.5297.221

[B3] Adler A. Zhao R. Shin M. E. Yasuda R. Gan W. B. (2019). Somatostatin-expressing interneurons enable and maintain learning-dependent sequential activation of pyramidal neurons. *Neuron* 102 202–216.e7. 10.1016/j.neuron.2019.01.036 30792151 PMC6555419

[B4] Almási Z. David C. Witte M. Staiger J. F. (2019). Distribution patterns of three molecularly defined classes of GABAergic neurons across columnar compartments in mouse barrel cortex. *Front. Neuroanat.* 13:45. 10.3389/fnana.2019.00045 31114486 PMC6503091

[B5] Apicella A. J. Marchionni I. (2022). VIP-expressing GABAergic neurons: Disinhibitory vs. Inhibitory motif and its role in communication across neocortical areas. *Front. Cell Neurosci.* 16:811484. 10.3389/fncel.2022.811484 35221922 PMC8867699

[B6] Artinian J. Lacaille J. C. (2018). Disinhibition in learning and memory circuits: New vistas for somatostatin interneurons and long-term synaptic plasticity. *Brain Res. Bull.* 141 20–26. 10.1016/j.brainresbull.2017.11.012 29174732

[B7] Ascoli G. A. Alonso-Nanclares L. Anderson S. A. Barrionuevo G. Benavides-Piccione R. Burkhalter A. (2008). Petilla terminology: Nomenclature of features of GABAergic interneurons of the cerebral cortex. *Nat. Rev. Neurosci.* 9 557–568. 10.1038/nrn2402 18568015 PMC2868386

[B8] Bi G. Q. Poo M. M. (1998). Synaptic modifications in cultured hippocampal neurons: Dependence on spike timing, synaptic strength, and postsynaptic cell type. *J. Neurosci.* 18 10464–10472. 9852584 10.1523/JNEUROSCI.18-24-10464.1998 PMC6793365

[B9] Bittner K. C. Milstein A. D. Grienberger C. Romani S. Magee J. C. (2017). Behavioral time scale synaptic plasticity underlies CA1 place fields. *Science* 357 1033–1036. 28883072 10.1126/science.aan3846 PMC7289271

[B10] Blackman A. V. Abrahamsson T. Costa R. P. Lalanne T. Sjöström P. J. (2013). Target cell-specific short-term plasticity in local circuits. *Front. Synapt. Neurosci.* 5:11. 10.3389/fnsyn.2013.00011 24367330 PMC3854841

[B11] Bliss T. V. Collingridge G. L. (1993). A synaptic model of memory: Long-term potentiation in the hippocampus. *Nature* 361 31–39. 8421494 10.1038/361031a0

[B12] Bredt D. S. Nicoll R. A. (2003). AMPA receptor trafficking at excitatory synapses. *Neuron* 40 361–379. 14556714 10.1016/s0896-6273(03)00640-8

[B13] Brock J. A. Thomazeau A. Watanabe A. Li S. S. Y. Sjöström P. J. (2020). A practical guide to using CV analysis for determining the locus of synaptic plasticity. *Front. Synapt. Neurosci.* 12:11. 10.3389/fnsyn.2020.00011 32292337 PMC7118219

[B14] Buchanan K. A. Blackman A. V. Moreau A. W. Elgar D. Costa R. P. Lalanne T. (2012). Target-specific expression of presynaptic NMDA receptors in neocortical microcircuits. *Neuron* 75 451–466. 10.1016/j.neuron.2012.06.017 22884329 PMC3657167

[B15] Camiré O. Topolnik L. (2014). Dendritic calcium nonlinearities switch the direction of synaptic plasticity in fast-spiking interneurons. *J. Neurosci.* 34 3864–3877. 10.1523/JNEUROSCI.2253-13.2014 24623765 PMC6705275

[B16] Campagnola L. Seeman S. C. Chartrand T. Kim L. Hoggarth A. Gamlin C. (2022). Local connectivity and synaptic dynamics in mouse and human neocortex. *Science* 375:eabj5861. 10.1126/science.abj5861 PMC9970277 35271334

[B17] Castillo P. E. Younts T. J. Chavez A. E. Hashimotodani Y. (2012). Endocannabinoid signaling and synaptic function. *Neuron* 76 70–81. 23040807 10.1016/j.neuron.2012.09.020 PMC3517813

[B18] Chistiakova M. Ilin V. Roshchin M. Bannon N. Malyshev A. Kisvarday Z. (2019). Distinct heterosynaptic plasticity in fast spiking and non-fast-spiking inhibitory neurons in rat visual cortex. *J. Neurosci.* 39 6865–6878. 10.1523/JNEUROSCI.3039-18.2019 31300522 PMC6733570

[B19] Chiu C. Q. Martenson J. S. Yamazaki M. Natsume R. Sakimura K. Tomita S. (2018). Input-specific NMDAR-dependent potentiation of dendritic GABAergic inhibition. *Neuron* 97 368-377.e3. 10.1016/j.neuron.2017.12.032 29346754 PMC5777295

[B20] Chou C. Y. C. Wong H. H. W. Guo C. Boukoulou K. E. Huang C. Jannat J. (2023). Principles of visual cortex excitatory microcircuit organization. *bioRxiv [Preprint]* 10.1101/2023.12.30.573666 PMC11763898 39872485

[B21] Cline H. T. (1998). Topographic maps: Developing roles of synaptic plasticity. *Curr. Biol.* 8 R836–R839. 9822571 10.1016/s0960-9822(07)00525-8

[B22] Costa R. P. Froemke R. C. Sjöström P. J. van Rossum M. C. W. (2015). Unified pre- and postsynaptic long-term plasticity enables reliable and flexible learning. *eLife* 4:e09457. 10.7554/eLife.09457 PMC4584257 26308579

[B23] Costa R. P. Mizusaki B. E. Sjöström P. J. van Rossum M. C. (2017). Functional consequences of pre- and postsynaptic expression of synaptic plasticity. *Philos. Trans. R. Soc. Lond. B Biol. Sci* 372:20160153. 10.1098/rstb.2016.0153 PMC5247585 28093547

[B24] Cunha-Reis D. Caulino-Rocha A. (2020). VIP modulation of hippocampal synaptic plasticity: A role for VIP receptors as therapeutic targets in cognitive decline and mesial temporal lobe epilepsy. *Front. Cell Neurosci.* 14:153. 10.3389/fncel.2020.00153 32595454 PMC7303298

[B25] D’Amour J. A. Froemke R. C. (2015). Inhibitory and excitatory spike-timing-dependent plasticity in the auditory cortex. *Neuron* 86 514–528. 25843405 10.1016/j.neuron.2015.03.014 PMC4409545

[B26] Debanne D. Gahwiler B. H. Thompson S. M. (1994). Asynchronous pre- and postsynaptic activity induces associative long-term depression in area CA1 of the rat hippocampus in vitro. *Proc. Natl. Acad. Sci. U. S. A.* 91 1148–1152. 7905631 10.1073/pnas.91.3.1148 PMC521471

[B27] Edelmann E. Cepeda-Prado E. Lessmann V. (2017). Coexistence of multiple types of synaptic plasticity in individual hippocampal CA1 pyramidal neurons. *Front. Synapt. Neurosci.* 9:7. 10.3389/fnsyn.2017.00007 28352224 PMC5348504

[B28] Egger V. Feldmeyer D. Sakmann B. (1999). Coincidence detection and changes of synaptic efficacy in spiny stellate neurons in rat barrel cortex. *Nat. Neurosci.* 2 1098–1105. 10.1038/16026 10570487

[B29] Feldman D. E. (2012). The spike-timing dependence of plasticity. *Neuron* 75 556–571. 22920249 10.1016/j.neuron.2012.08.001 PMC3431193

[B30] Field R. E. D’Amour J. A. Tremblay R. Miehl C. Rudy B. Gjorgjieva J. (2020). Heterosynaptic plasticity determines the set point for cortical excitatory-inhibitory balance. *Neuron* 106 842–854.e4. 10.1016/j.neuron.2020.03.002 32213321 PMC7274908

[B31] Fortune E. S. Rose G. J. (2001). Short-term synaptic plasticity as a temporal filter. *Trends Neurosci.* 24 381–385. 11410267 10.1016/s0166-2236(00)01835-x

[B32] Froemke R. C. Dan Y. (2002). Spike-timing-dependent synaptic modification induced by natural spike trains. *Nature* 416 433–438. 11919633 10.1038/416433a

[B33] Froemke R. C. Merzenich M. M. Schreiner C. E. (2007). A synaptic memory trace for cortical receptive field plasticity. *Nature* 450 425–429. 10.1038/nature06289 18004384

[B34] Froemke R. C. Tsay I. A. Raad M. Long J. D. Dan Y. (2006). Contribution of individual spikes in burst-induced long-term synaptic modification. *J. Neurophysiol.* 95 1620–1629. 10.1152/jn.00910.2005 16319206

[B35] Fu Y. Kaneko M. Tang Y. Alvarez-Buylla A. Stryker M. P. (2015). A cortical disinhibitory circuit for enhancing adult plasticity. *Elife* 4:e05558. 10.7554/eLife.05558 25626167 PMC4337686

[B36] Fu Y. Tucciarone J. M. Espinosa J. S. Sheng N. Darcy D. P. Nicoll R. A. (2014). A cortical circuit for gain control by behavioral state. *Cell* 156 1139–1152. 24630718 10.1016/j.cell.2014.01.050 PMC4041382

[B37] Fuhrmann G. Segev I. Markram H. Tsodyks M. (2002). Coding of temporal information by activity-dependent synapses. *J. Neurophysiol.* 87 140–148. 11784736 10.1152/jn.00258.2001

[B38] Gonchar Y. Wang Q. Burkhalter A. (2007). Multiple distinct subtypes of GABAergic neurons in mouse visual cortex identified by triple immunostaining. *Front. Neuroanat.* 1:3. 10.3389/neuro.05.003.2007 18958197 PMC2525923

[B39] Gouwens N. W. Sorensen S. A. Baftizadeh F. Budzillo A. Lee B. R. Jarsky T. (2020). Integrated morphoelectric and transcriptomic classification of cortical GABAergic cells. *Cell* 183 935–953.e19. 33186530 10.1016/j.cell.2020.09.057 PMC7781065

[B40] Hebb D. O. (1949). *The Organization of Behavior.* New York, NY: Wiley.

[B41] Holmgren C. D. Zilberter Y. (2001). Coincident spiking activity induces long-term changes in inhibition of neocortical pyramidal cells. *J. Neurosci.* 21 8270–8277. 10.1523/JNEUROSCI.21-20-08270.2001 11588198 PMC6763875

[B42] Holthoff K. Kovalchuk Y. Yuste R. Konnerth A. (2004). Single-shock LTD by local dendritic spikes in pyramidal neurons of mouse visual cortex. *J. Physiol.* 560 27–36. 10.1113/jphysiol.2004.072678 15319420 PMC1665193

[B43] Inagaki T. Begum T. Reza F. Horibe S. Inaba M. Yoshimura Y. (2008). Brain-derived neurotrophic factor-mediated retrograde signaling required for the induction of long-term potentiation at inhibitory synapses of visual cortical pyramidal neurons. *Neurosci. Res.* 61 192–200. 10.1016/j.neures.2008.02.006 18395922

[B44] Kaneko M. Stryker M. P. (2014). Sensory experience during locomotion promotes recovery of function in adult visual cortex. *Elife* 3:e02798. 10.7554/eLife.02798 24970838 PMC4070284

[B45] Katz L. C. Shatz C. J. (1996). Synaptic activity and the construction of cortical circuits. *Science* 274 1133–1138. 8895456 10.1126/science.274.5290.1133

[B46] Kepecs A. Fishell G. (2014). Interneuron cell types are fit to function. *Nature* 505 318–326. 24429630 10.1038/nature12983 PMC4349583

[B47] Kessels H. W. Malinow R. (2009). Synaptic AMPA receptor plasticity and behavior. *Neuron* 61 340–350. 19217372 10.1016/j.neuron.2009.01.015 PMC3917551

[B48] Khoshkhoo S. Vogt D. Sohal V. S. (2017). Dynamic, cell-type-specific roles for GABAergic interneurons in a mouse model of optogenetically inducible seizures. *Neuron* 93 291–298. 10.1016/j.neuron.2016.11.043 28041880 PMC5268075

[B49] Kullander K. Topolnik L. (2021). Cortical disinhibitory circuits: Cell types, connectivity and function. *Trends Neurosci.* 44 643–657. 34006387 10.1016/j.tins.2021.04.009

[B50] Lalanne T. Abrahamsson T. Sjöström P. J. (2016). Using multiple whole-cell recordings to study spike-timing-dependent plasticity in acute neocortical slices. *Cold Spring Harb. Protoc.* 2016:pdb prot091306. 10.1101/pdb.prot091306 27250948 PMC5298902

[B51] Larkum M. (2013). A cellular mechanism for cortical associations: An organizing principle for the cerebral cortex. *Trends Neurosci.* 36 141–151. 23273272 10.1016/j.tins.2012.11.006

[B52] Larsen R. S. Sjöström P. J. (2015). Synapse-type-specific plasticity in local circuits. *Curr. Opin. Neurobiol.* 35 127–135. 26310110 10.1016/j.conb.2015.08.001 PMC5280068

[B53] Lein E. S. Hawrylycz M. J. Ao N. Ayres M. Bensinger A. Bernard A. (2007). Genome-wide atlas of gene expression in the adult mouse brain. *Nature* 445 168–176. 17151600 10.1038/nature05453

[B54] Leroy F. de Solis C. A. Boyle L. M. Bock T. Lofaro O. M. Buss E. W. (2022). Enkephalin release from VIP interneurons in the hippocampal CA2/3a region mediates heterosynaptic plasticity and social memory. *Mol. Psychiatry* 27 2879–2900. 10.1038/s41380-021-01124-y 33990774 PMC8590711

[B55] Letzkus J. J. Wolff S. B. Luthi A. (2015). Disinhibition, a circuit mechanism for associative learning and memory. *Neuron* 88 264–276. 26494276 10.1016/j.neuron.2015.09.024

[B56] Letzkus J. J. Wolff S. B. Meyer E. M. Tovote P. Courtin J. Herry C. (2011). A disinhibitory microcircuit for associative fear learning in the auditory cortex. *Nature* 480 331–335. 10.1038/nature10674 22158104

[B57] Lisman J. Spruston N. (2005). Postsynaptic depolarization requirements for LTP and LTD: A critique of spike timing-dependent plasticity. *Nat. Neurosci.* 8 839–841. 10.1038/nn0705-839 16136666

[B58] Lisman J. Spruston N. (2010). Questions About STDP as a general model of synaptic plasticity. *Front. Synapt. Neurosci.* 2:140. 10.3389/fnsyn.2010.00140 21423526 PMC3059684

[B59] Lowel S. Singer W. (1992). Selection of intrinsic horizontal connections in the visual cortex by correlated neuronal activity. *Science* 255 209–212. 1372754 10.1126/science.1372754

[B60] Lu J. T. Li C. Y. Zhao J. P. Poo M. M. Zhang X. H. (2007). Spike-timing-dependent plasticity of neocortical excitatory synapses on inhibitory interneurons depends on target cell type. *J. Neurosci.* 27 9711–9720. 17804631 10.1523/JNEUROSCI.2513-07.2007 PMC6672961

[B61] Maass W. Zador A. M. (1999). Dynamic stochastic synapses as computational units. *Neural Comput.* 11 903–917. 10226188 10.1162/089976699300016494

[B62] Maffei A. Nataraj K. Nelson S. B. Turrigiano G. G. (2006). Potentiation of cortical inhibition by visual deprivation. *Nature* 443 81–84. 16929304 10.1038/nature05079

[B63] Malenka R. C. Bear M. F. (2004). LTP and LTD: An embarrassment of riches. *Neuron* 44 5–21. 15450156 10.1016/j.neuron.2004.09.012

[B64] Malinow R. Malenka R. C. (2002). AMPA receptor trafficking and synaptic plasticity. *Annu. Rev. Neurosci.* 25 103–126. 12052905 10.1146/annurev.neuro.25.112701.142758

[B65] Markram H. Gerstner W. Sjöström P. J. (2011). A history of spike-timing-dependent plasticity. *Front. Synapt. Neurosci.* 3:4. 10.3389/fnsyn.2011.00004 22007168 PMC3187646

[B66] Markram H. Gerstner W. Sjöström P. J. (2012). Spike-timing-dependent plasticity: A comprehensive overview. *Front. Synapt. Neurosci.* 4:2. 10.3389/fnsyn.2012.00002 22807913 PMC3395004

[B67] Markram H. Lübke J. Frotscher M. Sakmann B. (1997). Regulation of synaptic efficacy by coincidence of postsynaptic APs and EPSPs. *Science* 275 213–215. 10.1126/science.275.5297.213 8985014

[B68] Markram H. Tsodyks M. (1996). Redistribution of synaptic efficacy between neocortical pyramidal neurons. *Nature* 382 807–810. 8752273 10.1038/382807a0

[B69] Matveev V. Wang X. J. (2000). Differential short-term synaptic plasticity and transmission of complex spike trains: To depress or to facilitate? *Cereb. Cortex* 10 1143–1153. 11053234 10.1093/cercor/10.11.1143

[B70] McFarlan A. R. Chou C. Y. C. Watanabe A. Cherepacha N. Haddad M. Owens H. (2023). The plasticitome of cortical interneurons. *Nat. Rev. Neurosci.* 24 80–97. 10.1038/s41583-022-00663-9 36585520

[B71] Mizusaki B. E. P. Li S. S. Y. Costa R. P. Sjöström P. J. (2022). Pre- and postsynaptically expressed spike-timing-dependent plasticity contribute differentially to neuronal learning. *PLoS Comput. Biol.* 18:e1009409. 10.1371/journal.pcbi.1009409 35700188 PMC9236267

[B72] Muir J. Arancibia-Carcamo I. L. MacAskill A. F. Smith K. R. Griffin L. D. Kittler J. T. (2010). NMDA receptors regulate GABAA receptor lateral mobility and clustering at inhibitory synapses through serine 327 on the gamma2 subunit. *Proc. Natl. Acad. Sci. U. S. A.* 107 16679–16684. 10.1073/pnas.1000589107 20823221 PMC2944765

[B73] Myatt D. R. Hadlington T. Ascoli G. A. Nasuto S. J. (2012). Neuromantic - from semi-manual to semi-automatic reconstruction of neuron morphology. *Front. Neuroinform.* 6:4. 10.3389/fninf.2012.00004 22438842 PMC3305991

[B74] Myers-Joseph D. Wilmes K. A. Fernandez-Otero M. Clopath C. Khan A. G. (2023). Disinhibition by VIP interneurons is orthogonal to cross-modal attentional modulation in primary visual cortex. *Neuron* 112 628–645.e7. 10.1016/j.neuron.2023.11.006 38070500

[B75] Nabavi S. Fox R. Proulx C. D. Lin J. Y. Tsien R. Y. Malinow R. (2014). Engineering a memory with LTD and LTP. *Nature* 511 348–352. 24896183 10.1038/nature13294 PMC4210354

[B76] Nagel G. Szellas T. Huhn W. Kateriya S. Adeishvili N. Berthold P. (2003). Channelrhodopsin-2, a directly light-gated cation-selective membrane channel. *Proc. Natl. Acad. Sci. U. S. A.* 100 13940–13945. 14615590 10.1073/pnas.1936192100 PMC283525

[B77] Niell C. M. Stryker M. P. (2010). Modulation of visual responses by behavioral state in mouse visual cortex. *Neuron* 65 472–479. 10.1016/j.neuron.2010.01.033 20188652 PMC3184003

[B78] Obermayer J. Luchicchi A. Heistek T. S. de Kloet S. F. Terra H. Bruinsma B. (2019). Prefrontal cortical ChAT-VIP interneurons provide local excitation by cholinergic synaptic transmission and control attention. *Nat. Commun.* 10:5280. 10.1038/s41467-019-13244-9 PMC6872593 31754098

[B79] Ormond J. Woodin M. A. (2009). Disinhibition mediates a form of hippocampal long-term potentiation in area CA1. *PLoS One* 4:e7224. 10.1371/journal.pone.0007224 19787049 PMC2746290

[B80] Ormond J. Woodin M. A. (2011). Disinhibition-mediated LTP in the hippocampus is synapse specific. *Front. Cell Neurosci.* 5:17. 10.3389/fncel.2011.00017 21954377 PMC3175589

[B81] Pawlak V. Wickens J. R. Kirkwood A. Kerr J. N. D. (2010). Timing is not everything: Neuromodulation opens the STDP Gate. *Front. Synapt. Neurosci.* 2:146. 10.3389/fnsyn.2010.00146 21423532 PMC3059689

[B82] Pfeffer C. K. Xue M. He M. Huang Z. J. Scanziani M. (2013). Inhibition of inhibition in visual cortex: The logic of connections between molecularly distinct interneurons. *Nat. Neurosci.* 16 1068–1076. 10.1038/nn.3446 23817549 PMC3729586

[B83] Pfister J. P. Gerstner W. (2006). Triplets of spikes in a model of spike timing-dependent plasticity. *J. Neurosci.* 26 9673–9682. 16988038 10.1523/JNEUROSCI.1425-06.2006 PMC6674434

[B84] Piette C. Cui Y. Gervasi N. Venance L. (2020). Lights on endocannabinoid-mediated synaptic potentiation. *Front. Mol. Neurosci.* 13:132. 10.3389/fnmol.2020.00132 PMC7399367 32848597

[B85] Poncer J. C. Malinow R. (2001). Postsynaptic conversion of silent synapses during LTP affects synaptic gain and transmission dynamics. *Nat. Neurosci.* 4 989–996. 10.1038/nn719 11544481

[B86] Pouille F. Scanziani M. (2004). Routing of spike series by dynamic circuits in the hippocampus. *Nature* 429 717–723. 10.1038/nature02615 15170216

[B87] Prönneke A. Scheuer B. Wagener R. J. Möck M. Witte M. Staiger J. F. (2015). Characterizing VIP neurons in the barrel cortex of VIPcre/tdTomato mice reveals layer-specific differences. *Cereb. Cortex* 25 4854–4868. 10.1093/cercor/bhv202 26420784 PMC4635925

[B88] Remy S. Spruston N. (2007). Dendritic spikes induce single-burst long-term potentiation. *Proc. Natl. Acad. Sci. U. S. A.* 104 17192–17197. 17940015 10.1073/pnas.0707919104 PMC2040482

[B89] Ren C. Peng K. Yang R. Liu W. Liu C. Komiyama T. (2022). Global and subtype-specific modulation of cortical inhibitory neurons regulated by acetylcholine during motor learning. *Neuron* 110 2334–2350 e8. 10.1016/j.neuron.2022.04.031 35584693 PMC9308684

[B90] Rosenbaum R. Rubin J. Doiron B. (2012). Short term synaptic depression imposes a frequency dependent filter on synaptic information transfer. *PLoS Comput. Biol.* 8:e1002557. 10.1371/journal.pcbi.1002557 22737062 PMC3380957

[B91] Sarihi A. Mirnajafi-Zadeh J. Jiang B. Sohya K. Safari M. S. Arami M. K. (2012). Cell type-specific, presynaptic LTP of inhibitory synapses on fast-spiking GABAergic neurons in the mouse visual cortex. *J. Neurosci.* 32 13189–13199. 10.1523/JNEUROSCI.1386-12.2012 22993435 PMC6621461

[B92] Schindelin J. Arganda-Carreras I. Frise E. Kaynig V. Longair M. Pietzsch T. (2012). Fiji: An open-source platform for biological-image analysis. *Nat. Methods* 9 676–682. 10.1038/nmeth.2019 22743772 PMC3855844

[B93] Shatz C. J. (1992). The developing brain. *Sci. Am.* 267 60–67. 1502524 10.1038/scientificamerican0992-60

[B94] Sholl D. A. (1953). Dendritic organization in the neurons of the visual and motor cortices of the cat. *J. Anat.* 87 387–406. 13117757 PMC1244622

[B95] Silberberg G. Markram H. (2007). Disynaptic inhibition between neocortical pyramidal cells mediated by Martinotti cells. *Neuron* 53 735–746. 17329212 10.1016/j.neuron.2007.02.012

[B96] Sippy T. Yuste R. (2013). Decorrelating action of inhibition in neocortical networks. *J. Neurosci.* 33 9813–9830. 23739978 10.1523/JNEUROSCI.4579-12.2013 PMC3715137

[B97] Sjöström P. J. (2021). Grand challenge at the frontiers of synaptic neuroscience. *Front. Synapt. Neurosci.* 13 748937. 10.3389/fnsyn.2021.748937 34759809 PMC8575031

[B98] Sjöström P. J. Turrigiano G. G. Nelson S. B. (2001). Rate, timing, and cooperativity jointly determine cortical synaptic plasticity. *Neuron* 32 1149–1164. 10.1016/s0896-6273(01)00542-6 11754844

[B99] Sjöström P. J. Turrigiano G. G. Nelson S. B. (2003). Neocortical LTD via coincident activation of presynaptic NMDA and cannabinoid receptors. *Neuron* 39 641–654. 10.1016/s0896-6273(03)00476-8 12925278

[B100] Sjöström P. J. Turrigiano G. G. Nelson S. B. (2007). Multiple forms of long-term plasticity at unitary neocortical layer 5 synapses. *Neuropharmacology* 52 176–184. 10.1016/j.neuropharm.2006.07.021 16895733

[B101] Song S. Abbott L. F. (2001). Cortical development and remapping through spike timing-dependent plasticity. *Neuron* 32 339–350. 10.1016/s0896-6273(01)00451-2 11684002

[B102] Song S. Miller K. D. Abbott L. F. (2000). Competitive Hebbian learning through spike-timing-dependent synaptic plasticity. *Nat. Neurosci.* 3 919–926. 10966623 10.1038/78829

[B103] Speed A. Haider B. (2021). Probing mechanisms of visual spatial attention in mice. *Trends Neurosci.* 44 822–836. 10.1016/j.tins.2021.07.009 34446296 PMC8484049

[B104] Stent G. S. (1973). A physiological mechanism for Hebb’s postulate of learning. *Proc. Natl. Acad. Sci. U. S. A.* 70 997–1001. 4352227 10.1073/pnas.70.4.997 PMC433410

[B105] Taniguchi H. He M. Wu P. Kim S. Paik R. Sugino K. (2011). A resource of Cre driver lines for genetic targeting of GABAergic neurons in cerebral cortex. *Neuron* 71 995–1013. 10.1016/j.neuron.2011.07.026 21943598 PMC3779648

[B106] Tremblay R. Lee S. Rudy B. (2016). GABAergic interneurons in the neocortex: From cellular properties to circuits. *Neuron* 91 260–292. 27477017 10.1016/j.neuron.2016.06.033 PMC4980915

[B107] Udakis M. Pedrosa V. Chamberlain S. E. L. Clopath C. Mellor J. R. (2020). Interneuron-specific plasticity at parvalbumin and somatostatin inhibitory synapses onto CA1 pyramidal neurons shapes hippocampal output. *Nat. Commun.* 11:4395. 10.1038/s41467-020-18074-8 32879322 PMC7467931

[B108] Vickers E. D. Clark C. Osypenko D. Fratzl A. Kochubey O. Bettler B. (2018). Parvalbumin-interneuron output synapses show spike-timing-dependent plasticity that contributes to auditory map remodeling. *Neuron* 99:e726. 10.1016/j.neuron.2018.07.018 30078579

[B109] Vogels T. P. Sprekeler H. Zenke F. Clopath C. Gerstner W. (2011). Inhibitory plasticity balances excitation and inhibition in sensory pathways and memory networks. *Science* 334 1569–1573. 22075724 10.1126/science.1211095

[B110] Walker F. Mock M. Feyerabend M. Guy J. Wagener R. J. Schubert D. (2016). Parvalbumin- and vasoactive intestinal polypeptide-expressing neocortical interneurons impose differential inhibition on Martinotti cells. *Nat. Commun.* 7:13664. 10.1038/ncomms13664 27897179 PMC5141346

[B111] Wang Y. Toledo-Rodriguez M. Gupta A. Wu C. Silberberg G. Luo J. (2004). Anatomical, physiological and molecular properties of Martinotti cells in the somatosensory cortex of the juvenile rat. *J. Physiol.* 561 65–90. 10.1113/jphysiol.2004.073353 15331670 PMC1665344

[B112] Wong H. H. Rannio S. Jones V. Thomazeau A. Sjöström P. J. (2021). NMDA receptors in axons: There’s no coincidence. *J. Physiol.* 599 367–387. 10.1113/JP280059 33141440

[B113] Woodin M. A. Ganguly K. Poo M.-M. (2003). Coincident Pre- and postsynaptic activity modifies GABAergic synapses by postsynaptic changes in Cl- transporter activity. *Neuron* 39 807–820. 10.1016/s0896-6273(03)00507-5 12948447

[B114] Yazaki-Sugiyama Y. Kang S. Cateau H. Fukai T. Hensch T. K. (2009). Bidirectional plasticity in fast-spiking GABA circuits by visual experience. *Nature* 462 218–221. 10.1038/nature08485 19907494

[B115] Zhou L. Nho K. Haddad M. G. Cherepacha N. Tubeleviciute-Aydin A. Tsai A. P. (2021). Rare CASP6N73T variant associated with hippocampal volume exhibits decreased proteolytic activity, synaptic transmission defect, and neurodegeneration. *Sci. Rep.* 11:12695. 10.1038/s41598-021-91367-0 PMC8209045 34135352

[B116] Zucker R. S. Regehr W. G. (2002). Short-term synaptic plasticity. *Annu. Rev. Physiol.* 64 355–405. 11826273 10.1146/annurev.physiol.64.092501.114547

