# Supplementary material for: The spike-timing-dependent plasticity of VIP interneurons in motor cortex
Source: Front Cell Neurosci. 2024 Apr 19;18:1389094. doi: 10.3389/fncel.2024.1389094 (PMC11066220; doi:10.3389/fncel.2024.1389094)
Supplement: Supplementary file 1 [file Data_Sheet_1.pdf]

frontiers

Supplementary Material

The spike-timing-dependent plasticity of VIP interneurons in motor cortex

Amanda R. McFarlan[1,2], Connie Guo[1,2], Isabella Gomez[1], Chaim Weinerman[1], Tasha A. Liang[1], P. Jesper Sjöström[1*]

[1] Centre for Research in Neuroscience, BRaIN Program, Dept of Neurology and Neurosurgery, Research Institute of the McGill University Health Centre, Montreal General Hospital, Montreal, QC, Canada

[2] Integrated Program in Neuroscience, McGill University, Montreal, QC, Canada

*** Correspondence:** jesper.sjostrom@mcgill.ca

1 Supplementary Figures

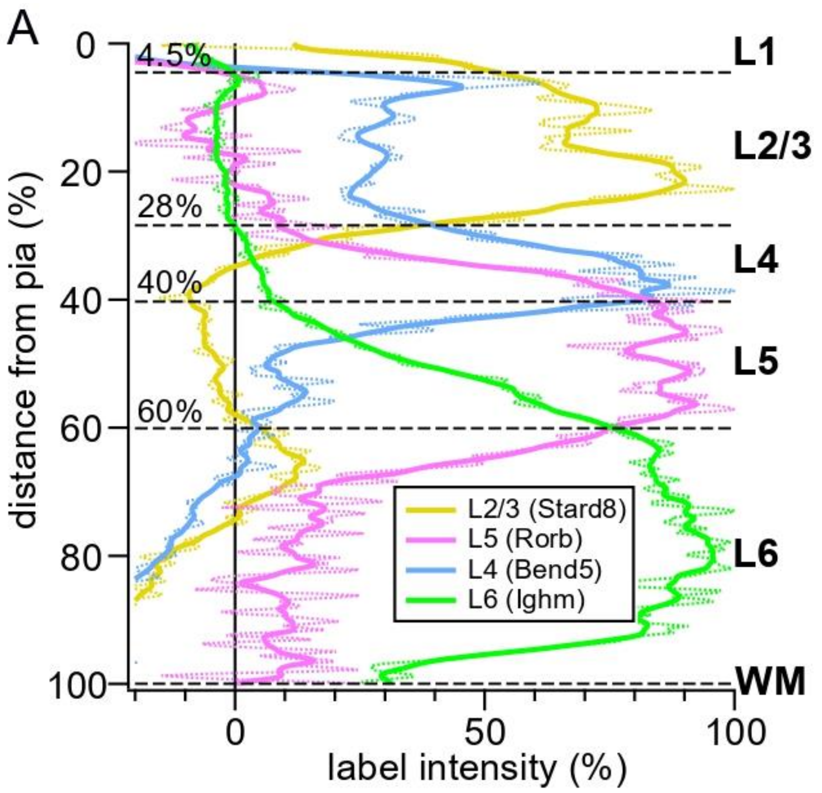

Supplementary Figure 1. Motor cortex layer boundaries were informed by genetic markers. (A) Whereas the boundaries to the virtually cell-free L1 and white-matter (WM) were trivial to define, boundaries between L2/3, L4, L5, and L6 were better demarcated using the differential expression of layer-specific genes Stard8, Rorb, Bend5, and Ighm, as retrieved from the Allen Institute Mouse Brain Atlas (Lein et al., 2007). The specific location of layer boundaries were thereby defined as the cross-over point of two adjacent label intensity profiles. The normalized distances from pial surface to WM (i.e., 4.5%, 28%, 40%, and 60%) were thus used as a reference to inform layer boundaries in individual slices. Dotted line: raw profile. Continuous line: box-smoothed profile. See Methods for details.

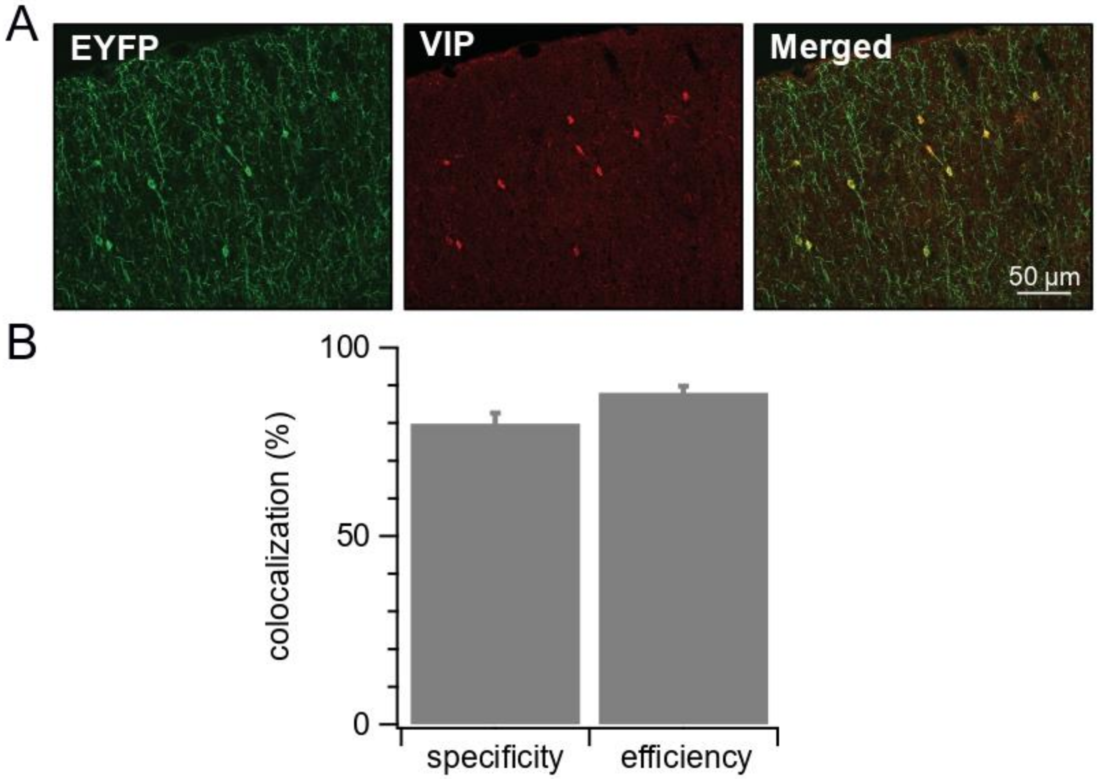

Supplementary Figure 2. ChR2 was targeted to VIP INs in the motor cortex. (A) 50-µm thick coronal sections from VIP-ChR2 mouse motor cortex were stained to tag EYFP-tagged ChR2-expressing cells ("EYFP"), and VIP-positive cells ("VIP"). The co-localization of EYFP and VIP is shown in the rightmost panel ("Merged"). **(B)** Quantification of the co-localization of VIP and EYFP in the VIP-ChR2 transgenic mice was performed across the entire cortical column and in both hemispheres (n = 26 sections, N = 8 animals). Specificity is defined as the percentage of EYFP-positive cells that are also VIP-positive, while efficiency is defined as the percentage of VIP-positive cells that are also EYFP-positive.

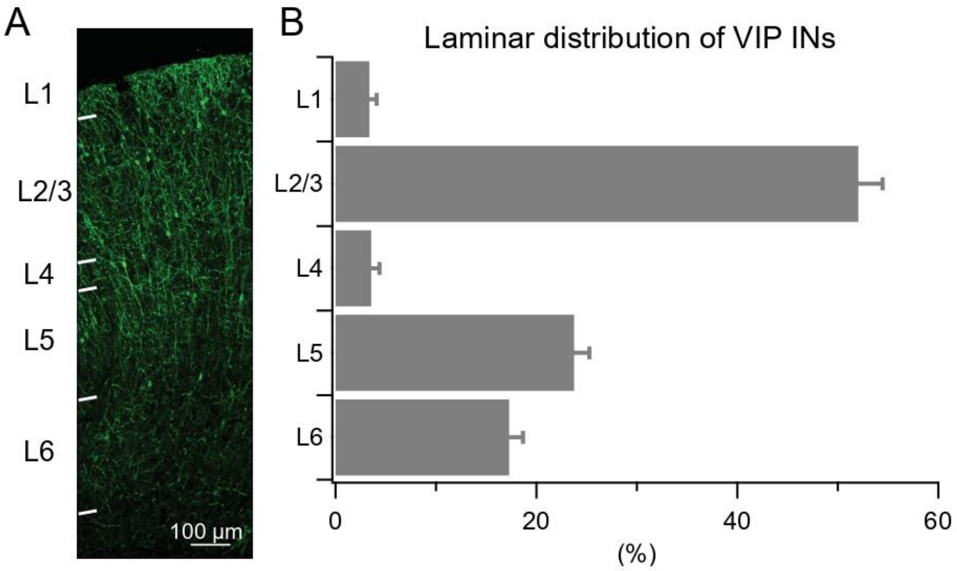

Supplementary Figure 3. VIP INs were most densely populated in L2/3 of the motor cortex. (A) Projection view of a 50-µm thick coronal section in the VIP-ChR2 transgenic mouse motor cortex. Sections were stained to tag ChR2-expressing VIP INs. **(B)** VIP INs most densely populated L2/3 of the motor cortex followed by L5, L6, L1, and L4 (n = 17 sections, N = 8 animals).

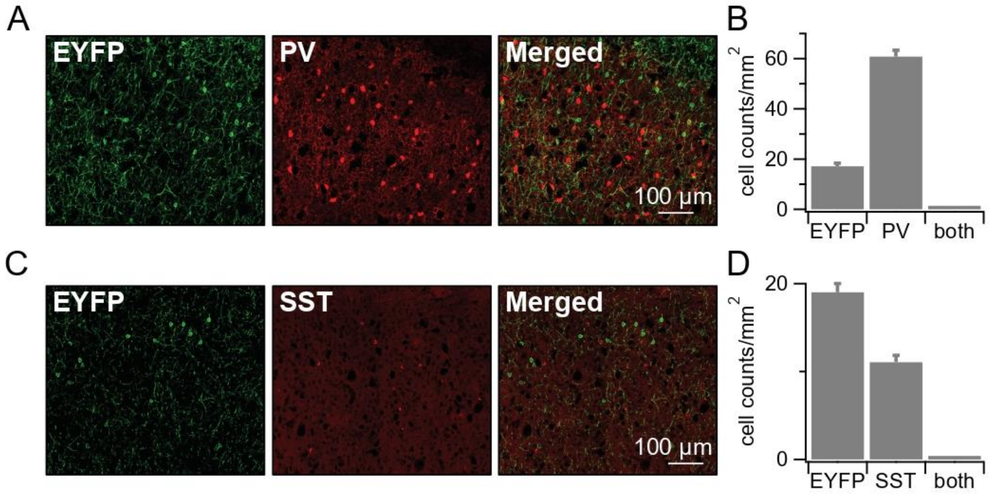

Supplementary Figure 4. VIP INs did not express PV or SST. (A) 50-µm thick coronal sections from VIP-ChR2 mouse motor cortex were stained to tag ChR2-expressing VIP INs ("EYFP"), and to tag PV-positive cells ("PV"). The co-localization of VIP INs and PV INs is shown in the rightmost panel ("Merged"). Cell counts were performed across the entire cortical column and in both hemispheres. **(B)** ChR2-expressing VIP INs (n = 26 sections, N = 5 animals) and PV-positive INs did not show any co-localization. **(C)** Same as (A), but sections were stained to tag ChR2-expressing VIP INs ("EYFP"), and SST-positive cells ("SST"). The co-localization of VIP INs and SST INs is shown in the rightmost panel ("Merged"). **(D)** ChR2-expressing VIP INs (n = 21 sections, N = 5 animals) and SST-positive INs did not show any co-localization.

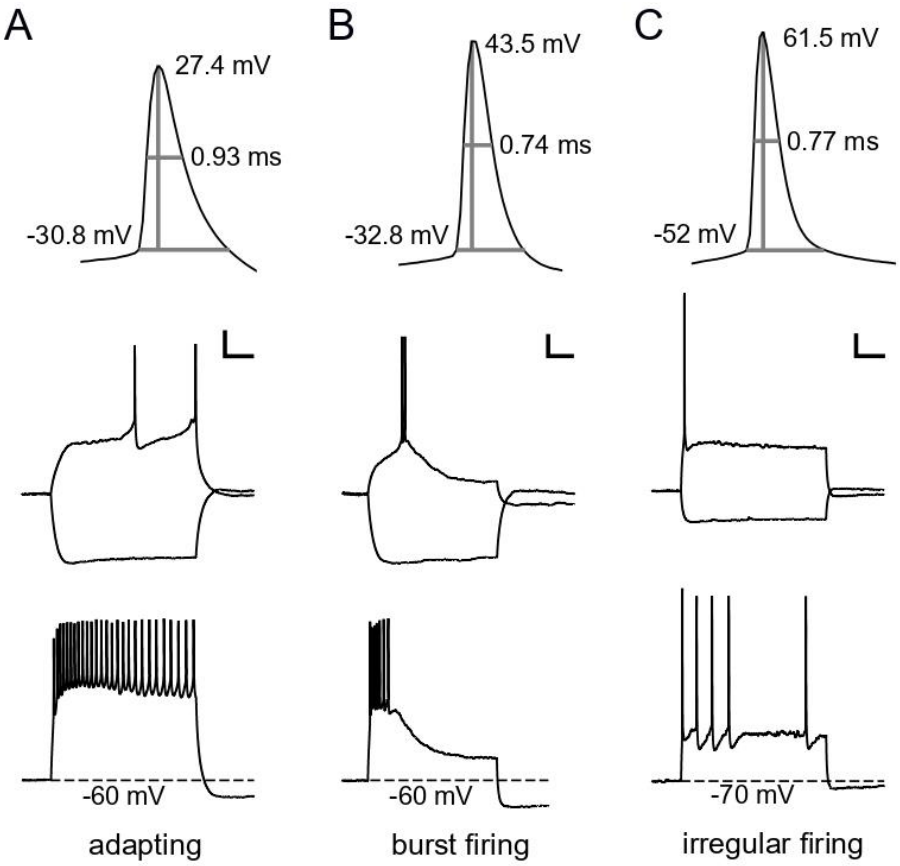

Supplementary Figure 5. VIP INs exhibited varying spike patterns. Sample electrophysiological traces of VIP INs with varying spike patterns. **(A)** (Top) zoomed in VIP IN action potential, (middle) VIP IN spiking at rheobase, and (bottom) VIP IN spiking at high frequency stimulation with adapting firing pattern. Dashed line indicates resting membrane potential. Scale bar is 100 ms on the x-axis, 10 mV on the y-axis. **(B)** Same as in (A), but for a VIP IN with a burst firing pattern. **(C)** Same as in (A), but for a VIP IN with an irregular firing pattern.

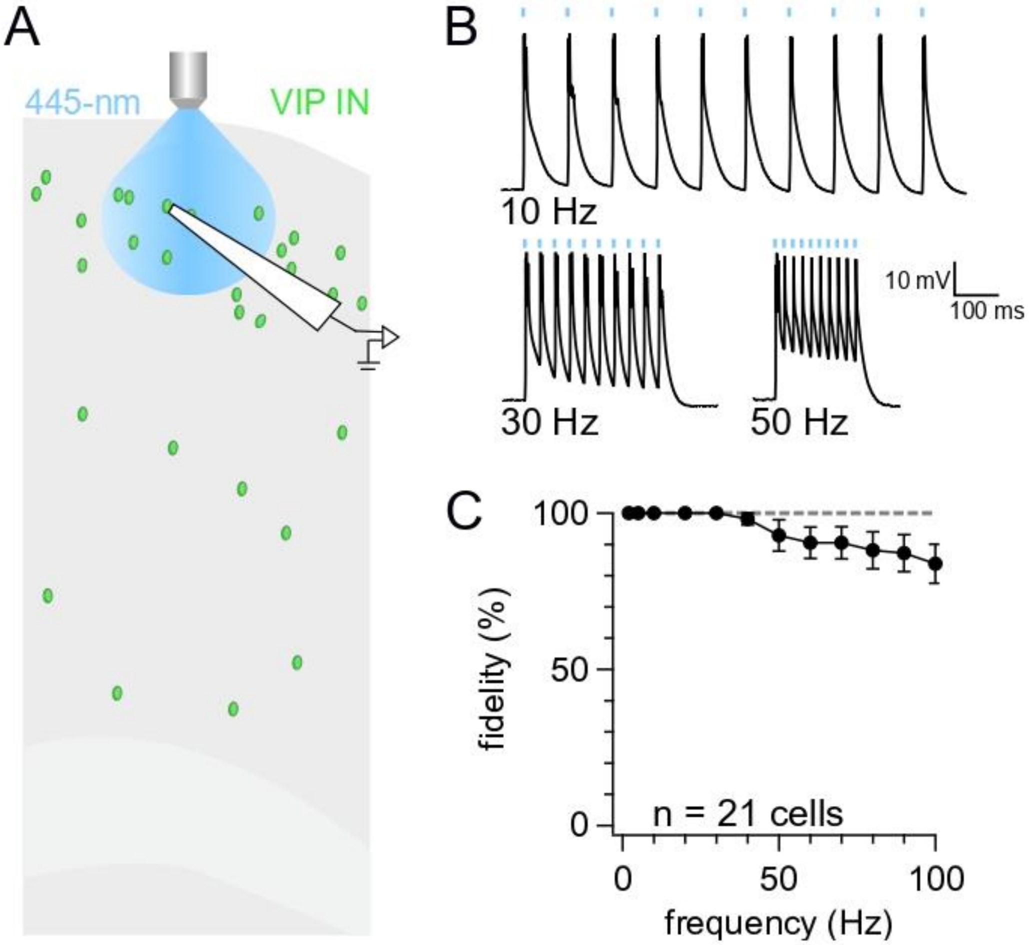

Supplementary Figure 6. ChR2-expressing VIP INs were reliably driven at 50 Hz. (A) Schematic of experimental paradigm. L2/3 VIP INs were targeted for whole-cell recording and activated with 445-nm blue laser light. **(B)** Representative spike trains in response to 10, 30 and 50 Hz blue light stimulation (blue lines) recorded from motor cortex VIP INs in current clamp. **(C)** Spiking was reliably evoked at stimulation frequencies up to 50 Hz (93% ± 5% fidelity, n = 21 cells, N = 12 animals).

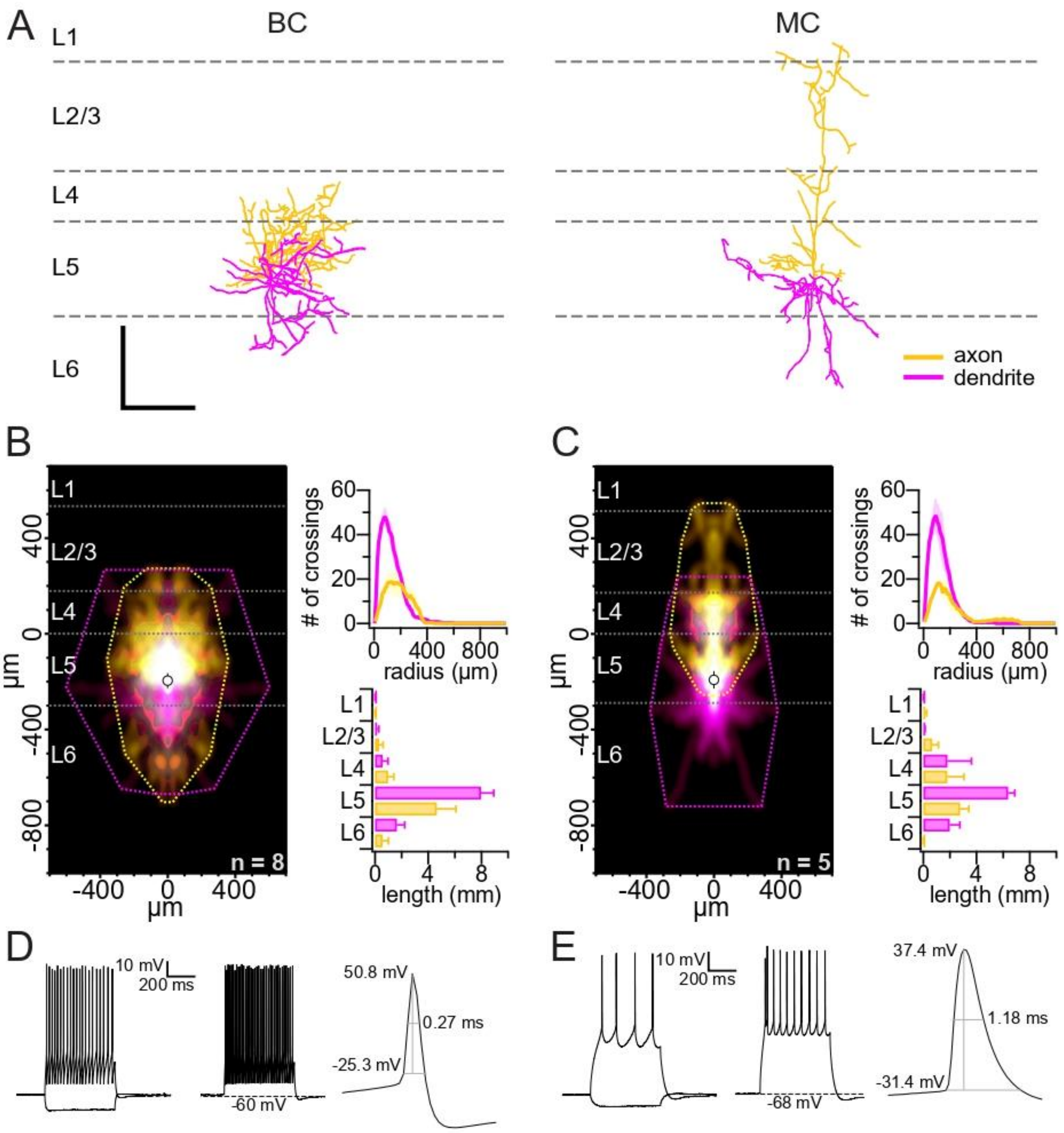

Supplementary Figure 7. Identification of L5 BCs and MCs in the motor cortex. (A) Sample reconstructions from a L5 BC (left) and from a L5 MC (right). Axons are labeled yellow and dendrites are labeled pink. Scale bar is 250 µm for both axes. Compartment density heat map (left), Sholl analysis (top right), and layer-specific branching (bottom right) for an arbitrary subset of **(B)** L5 BCs (n = 8 cells, N = 8 animals) and **(C)** L5 MCs (n = 5 cells, N = 5 animals) highlighted how MCs had ascending axons and dangling dendrites, while BCs had more locally branching axons and dendrites. In many cases, however, the MC ascending axon was accidentally cut during dissection. **(D)** Sample electrophysiology traces from a L5 BC revealed non-accommodating fast spiking at rheobase (left) as well as at stronger current injection (center), with characteristic sub-millisecond action potential half width (right). Dashed line indicates resting membrane potential. **(E)** The equivalent sample traces for an L5 MC revealed characteristic accommodating firing pattern with spike half width close to a millisecond.

2 Supplementary Tables

Supplementary Table 1. Motor cortex VIP IN electrophysiological properties did not differ. Properties were measured as indicated in Supplementary Figure 4. All p-values were obtained with ANOVA across L2/3, L5, and L6, except for firing pattern comparisons, for which Kruskal-Wallis was used.

Property	L2/3	L5	L6	p-value	Ensemble
Resting potential (mV)	-64 ± 2	-62 ± 4	-56 ± 7	0.31	-63 ± 2
Spike threshold (mV)	-40 ± 2	-34 ± 2	-38 ± 4	0.059	-38 ± 1
Spike height (mV)	46 ± 3	38 ± 4	49 ± 5	0.22	44 ± 2
Spike half width (ms)	0.93 ± 0.05	1.0 ± 0.08	1.0 ± 0.2	0.67	0.95 ± 0.04
Rheobase (pA)	75 ± 10	81 ± 20	53 ± 20	0.77	74 ± 10
Membrane time constant (ms)	18 ± 1	20 ± 2	26 ± 7	0.41	19 ± 1
Input resistance (MΩ)	273 ± 19	280 ± 36	311 ± 63	0.82	278 ± 16
Spike patterns:					
Adapting	13 (43%)	11 (92%)	3 (75%)	< 0.05	27 (59%)
Bursting	10 (33%)	1 (8%)	0 (0%)		11 (24%)
Irregular	7 (23%)	0 (0%)	1 (25%)		8 (17%)
n	30	12	4	-	46

Supplementary Table 2. BCs and MCs in L5 of motor cortex had distinct electrophysiological properties. Properties were measured as indicated in Supplementary Figure 6. All p-values were obtained with Wilcoxon-Mann-Whitney two-sample rank test, except for resting potential and spike threshold, for which Student's t-test was used.

Property	BC	MC	p-value
Resting potential (mV)	-63 ± 1	-62 ± 1	0.21
Spike threshold (mV)	-38 ± 1	-38 ± 1	0.99
Spike height (mV)	29 ± 1	38 ± 2	<0.01
Spike half width (ms)	0.43 ± 0.01	1.0 ± 0.04	<0.001
Rheobase (pA)	380 ± 20	130 ± 10	<0.001
Membrane time constant (ms)	10 ± 1	23 ± 1	<0.001
Input resistance (MΩ)	98 ± 6	190 ± 9	<0.001
n	61	96	-
